# Redirection of sphingolipid metabolism drives cytoskeletal defects in SPLIS and reveals ROCK inhibition as therapy

**DOI:** 10.1172/JCI194427

**Published:** 2026-04-23

**Authors:** Adam Majcher, Ranjha Khan, Kathrin Buder, Florence Bourquin, Julie D. Saba, Thorsten Hornemann

**Affiliations:** 1 Institute of Clinical Chemistry, University Hospital and University Zurich, Zurich, Switzerland.; 2 Department of Pediatrics, University of California San Francisco, California, USA.; 3 Department of General Pediatrics and Hematology/Oncology, University Hospital Tübingen, Tübingen, Germany.; 4 Department of Pediatric Nephrology, University Children`s Hospital Zürich, Zürich, Switzerland.

**Keywords:** Genetics, Metabolism, Nephrology, Chronic kidney disease, Cytoskeleton, Lipidomics

## Abstract

Sphingosine-1-phosphate lyase (SPL) insufficiency syndrome (SPLIS), also known as nephrotic syndrome type 14, is an autosomal recessive multisystem disorder caused by loss-of-function mutations in *SGPL1*, encoding the enzyme responsible for the terminal degradation of sphingosine-1-phosphate (S1P). We investigated a patient carrying a previously undescribed c.1084T>A (p.Ser362Thr) *SGPL1* variant and analyzed the metabolic and cellular consequences of SPL deficiency, using patient fibroblasts, *SGPL1*-KO HEK293T cells, and *Sgpl1^–/–^* and *Sgpl1^rosa+fl/fl^* mice. Metabolic stable isotope labeling revealed that SPL deficiency does not invariably result in S1P accumulation. Instead, SPL-deficient cells maintain near-normal S1P levels through (a) feedback regulation of de novo sphingolipid synthesis via the ORMDL–ceramide axis and (b) increased diversion of excess ceramides into glycosphingolipids. However, perturbation of sphingolipid homeostasis, either by exogenous sphingolipid load or disruption of compensatory regulation, induces pathological intracellular S1P accumulation. In vivo, *Sgpl1*^–/–^ mice had pronounced urinary S1P excretion and renal S1P enrichment, accompanied by cytoskeletal disorganization and impaired epithelial morphogenesis. Mechanistically, we identify aberrant Rho/ROCK signaling as a key mediator of S1P-driven cytoskeletal dysregulation. Pharmacological ROCK inhibition with fasudil mitigated renal cytoskeletal defects in *Sgpl1^–/–^* and *Sgpl1^rosa+fl/fl^* mice and partially restored epithelial architecture. These findings redefine the metabolic consequences of SPL deficiency and identify S1P-driven Rho/ROCK hyperactivation as a tractable therapeutic target in SPLIS.

## Abstract 2

We demonstrated that a toxic S1P buildup disrupts kidney cells in the genetic disease SPLIS The damage can be prevented by the approved drug Fasudil.

## Graphical abstract

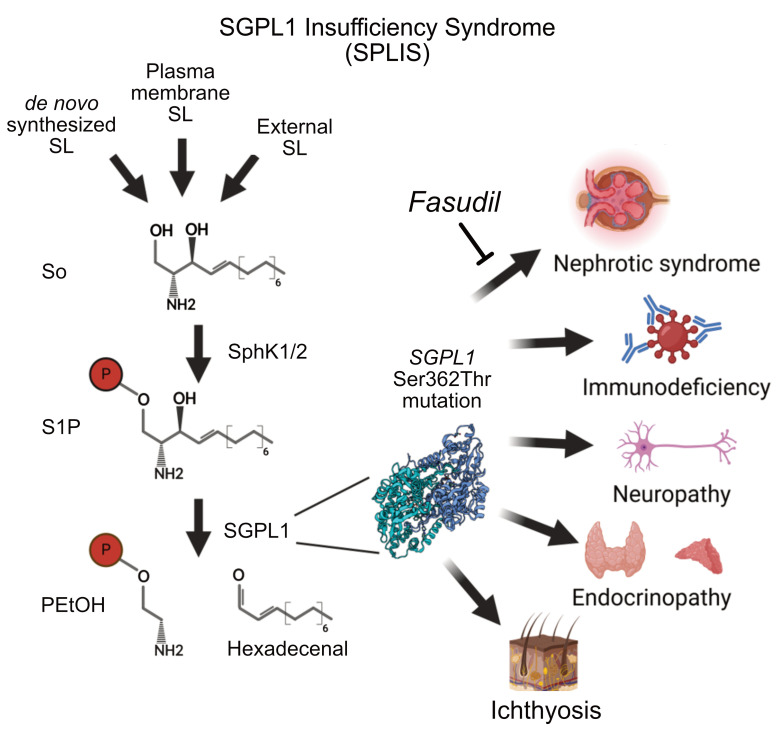

## Introduction

Sphingosine (So) and its phosphorylated derivative sphingosine-1-phosphate (S1P) are critical bioactive lipids involved in numerous physiological and pathological processes. S1P is a potent lipid signaling molecule critical for regulating immune cell trafficking, vascular development, and maintaining endothelial integrity (1). S1P exerts its effects primarily through G protein–coupled receptors (S1PRs), which include 5 distinct subtypes (S1PR1–S1PR5), each with specific expression patterns and functions (1). In addition to its receptor-dependent actions, S1P also functions in a receptor-independent manner, influencing intracellular calcium homeostasis (2) and mitochondrial function (3). The terminal degradation of S1P is mediated by sphingosine-1-phosphate lyase (SPL), which breaks it down into a long-chain aldehyde and phosphoethanolamine (4). SPL is encoded by the *SGPL1* gene and is localized to the endoplasmic reticulum, where it catalyzes the final step in the catabolism of sphingolipids (SLs).

SPL insufficiency syndrome (SPLIS), or nephrotic syndrome type 14 (also known as RENI syndrome), is a rare autosomal recessive disorder arising from biallelic mutations in *SGPL1* (5). Thus far, *SGPL1* mutations have been reported in more than 76 patients (6). The disease is characterized by immunodeficiency as well as renal, neurological, skin, and endocrine complications (7, 8). The overall mortality rate among patients with SPLIS is 50%, primarily within the first decade of life, often due to end-stage kidney disease and sepsis (6).

Previous studies in cellular and mouse models have shown that SPLIS-associated SPL variants have reduced activity, which results in increased S1P levels (9–11), suggesting that an aberrant S1P metabolism might contribute to the complications in SPLIS.

Serine palmitoyl transferase (SPT) catalyzes the first and rate-limiting step of SL de novo formation, opposing SPL activity. The SPT reaction typically conjugates l-serine and palmitoyl-CoA, forming the sphingoid base (keto) sphinganine (Sa), which is metabolized over several steps into ceramides (Cers) and complex SLs (4, 12). In addition to de novo synthesis as a source of SLs, cells and tissues can also absorb free and phosphorylated sphingoid bases from extracellular sources. Cellular uptake of S1P and other phosphorylated sphingoid bases requires their dephosphorylation, catalyzed by phospholipid phosphatases, followed by the passive absorption of the formed So (13). Additionally, S1P can enter the cell through S1P receptor uptake. The resorbed So is then either reacylated to form Cer (salvage pathway) or rephosphorylated to S1P. Additionally, S1P is exported via sphingolipid transporter 2 or by the major facilitator superfamily domain containing 2B, which are expressed in different cells (14).

However, because SPL is ubiquitously expressed in all cells and tissues except erythrocytes and platelets, it is surprising that a systemic mutation in a universally active metabolic pathway manifests predominantly as a severe renal phenotype. In this study, we investigated why the reduction in SPL activity primarily affects kidney function. Specifically, we sought to identify the mechanisms that make kidney cells particularly vulnerable in SPLIS and to determine whether these underlying mechanisms can be targeted therapeutically.

## Results

### A novel SPLIS-associated SGPL1p.(Ser362Thr) variant.

**Figure 1:**
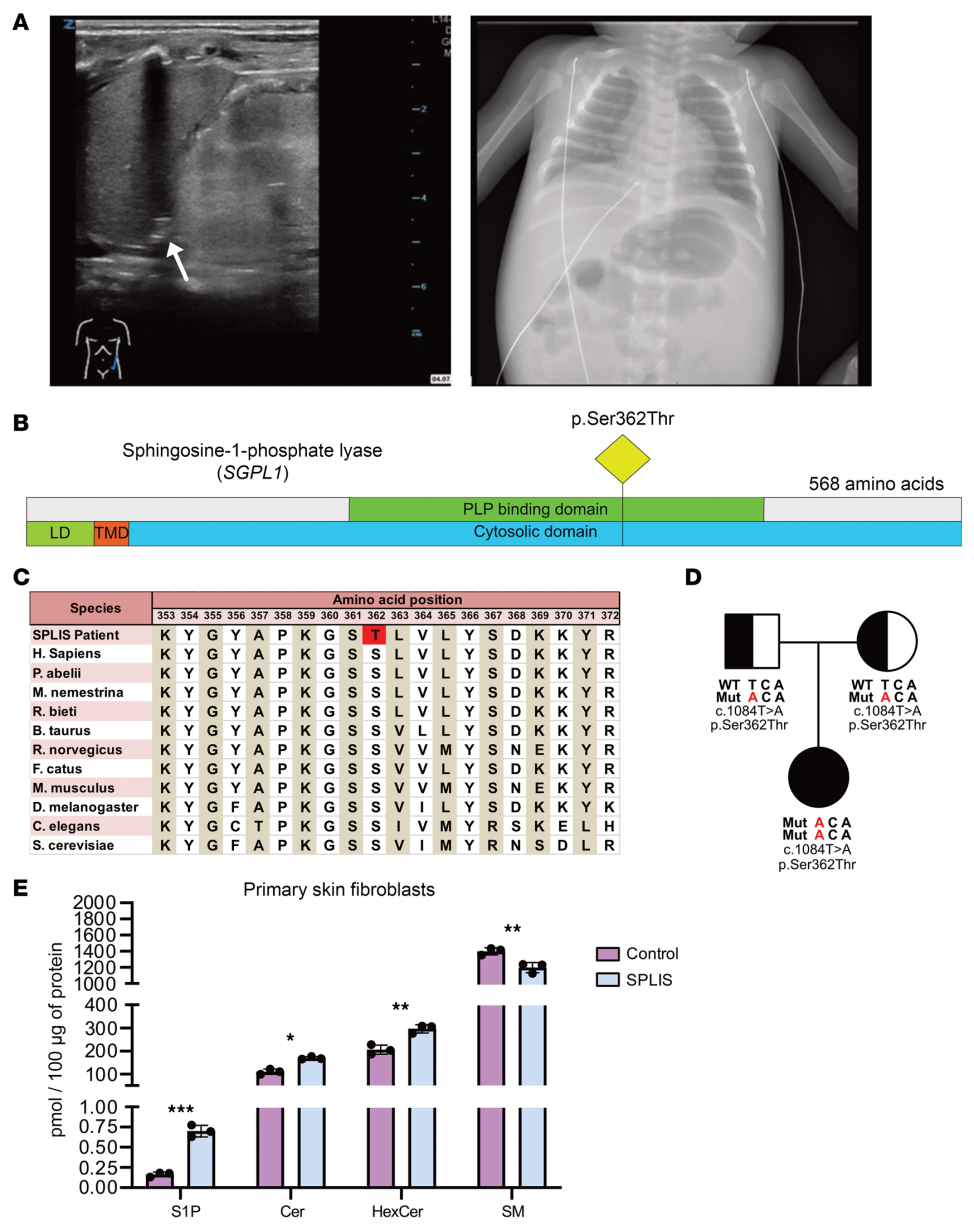
Clinical and molecular characterization of the new SPLIS-causing *SGPL1* mutation p.Ser362Thr. (**A**) Sonographic and X-ray imaging of a patient with the *SGPL1* mutation (p.Ser362Thr). Sonographic image shows a hyperechogenic kidney and adrenal calcifications (arrow). Only the left kidney and adrenal gland are depicted. The X-ray image reveals adrenal calcifications. (**B**) Schematic representation of the human *SGPL1* gene with an up-to-scale domain architecture. The SPLIS-associated mutation is marked with a yellow rectangle. (**C**) Conservation analysis of amino acid residues surrounding the SPLIS-causing mutation in *SGPL1*. The patient mutation (p.Ser362Thr) is highlighted in red. (**D**) Pedigree of the affected family, illustrating the segregation of the *SGPL1* variant (*NM_003901.3* c.1084T>A, p.Ser362Thr, Chr10(hg19):g.72633132T>A). (**E**) SL profile of primary skin fibroblasts from the patient with SPLIS versus from healthy control individuals (*n* = 3). Bar plots represent the mean ± SD of sums for each SL class. Statistical significance was assessed using a *t* test, with multiple testing correction applied via the 2-stage step-up method (Benjamini, Krieger, and Yekutieli). *q < 0.05, **q < 0.005, ***q < 0.0005.

An 8-week-old girl, a term firstborn of healthy, Iraqi, consanguineous parents (oligohydramnios, small-for-gestational age with birth weight of 2,140 g at 37+1 gestational weeks), was diagnosed with dialysis-dependent end-stage kidney disease due to congenital nephrotic syndrome presenting with heavy proteinuria, hypoalbuminemia, and edema. Kidney biopsy and steroid treatment were not performed, considering the patient’s young age, which was suggestive of a genetic cause of the nephrotic syndrome. Whole exome sequencing identified a homozygous variant in exon 12 of the *SGPL1* gene (c.1084T>A, p.Ser362Th) suspected to be pathogenic and causing SPLIS (Figure 1, B–D) (6). In the protein sequence, the mutation is located close to the cofactor-binding lysine at K353. Besides the renal phenotype, the patient exhibited multiple extrarenal manifestations (summarized in Table 1). Adrenal calcifications identified initially are shown in Figure 1A. The clinical course was complicated by prolonged hospitalizations due to recurrent respiratory tract infections with global respiratory insufficiency, recurrent cytomegalovirus viremia, and coagulase-negative staphylococci bacteremia, life-threatening arterial hypotension, catheter-related thromboses of several central veins of the upper thoracic aperture, and increased need for peritoneal dialysis due to ultrafiltration insufficiency. At 8 months, cerebral computed tomography performed after clinical presentation of a generalized seizure, showed slightly expanded cerebrospinal fluid spaces. The patient died at the age of 9 months, most likely due to septic shock with subsequent multiorgan failure.

To investigate whether the new *SGPL1* variant was associated with changes in overall plasma SL profile, we performed a lipidomics analysis, based on untargeted liquid chromatography–tandem mass spectrometry (LC-MS/MS), of the patient’s plasma (*n* = 1) compared with healthy control samples (*n* = 7). In our analysis, we did not see significantly elevated S1P levels, whereas Cer, hexosylceramide (HexCer) and sphingomyelin (SM) levels were slightly increased in the patient’s plasma (Supplemental Figure 1; supplemental material available online with this article; https://doi.org/10.1172/JCI194427DS1). However, due to the patient’s very young age and severe condition, only a limited amount of plasma was available. As a result, S1P levels were quantified from the general lipidomics analysis, which is not the best-suited method to measure phosphorylated sphingoid bases. Unfortunately, there was insufficient plasma to verify S1P levels through a separate, more sensitive, targeted S1P analysis. Patient-derived skin fibroblasts were significantly increased in S1P, Cer, and HexCer, whereas total SM was decreased (Figure 1E). However, the absolute increase in S1P was small (0.005 pmol/μg protein) compared with the total differences observed for HexCer (1 pmol/μg protein) or SM (2 pmol/μg protein). A similar change in the SL profile was also seen in HEK293T-*SGPL1* KO cells (data not shown).

### Downregulation of SL de novo synthesis prevents S1P accumulation in SPL/SPL-deficient cells.

**Figure 2:**
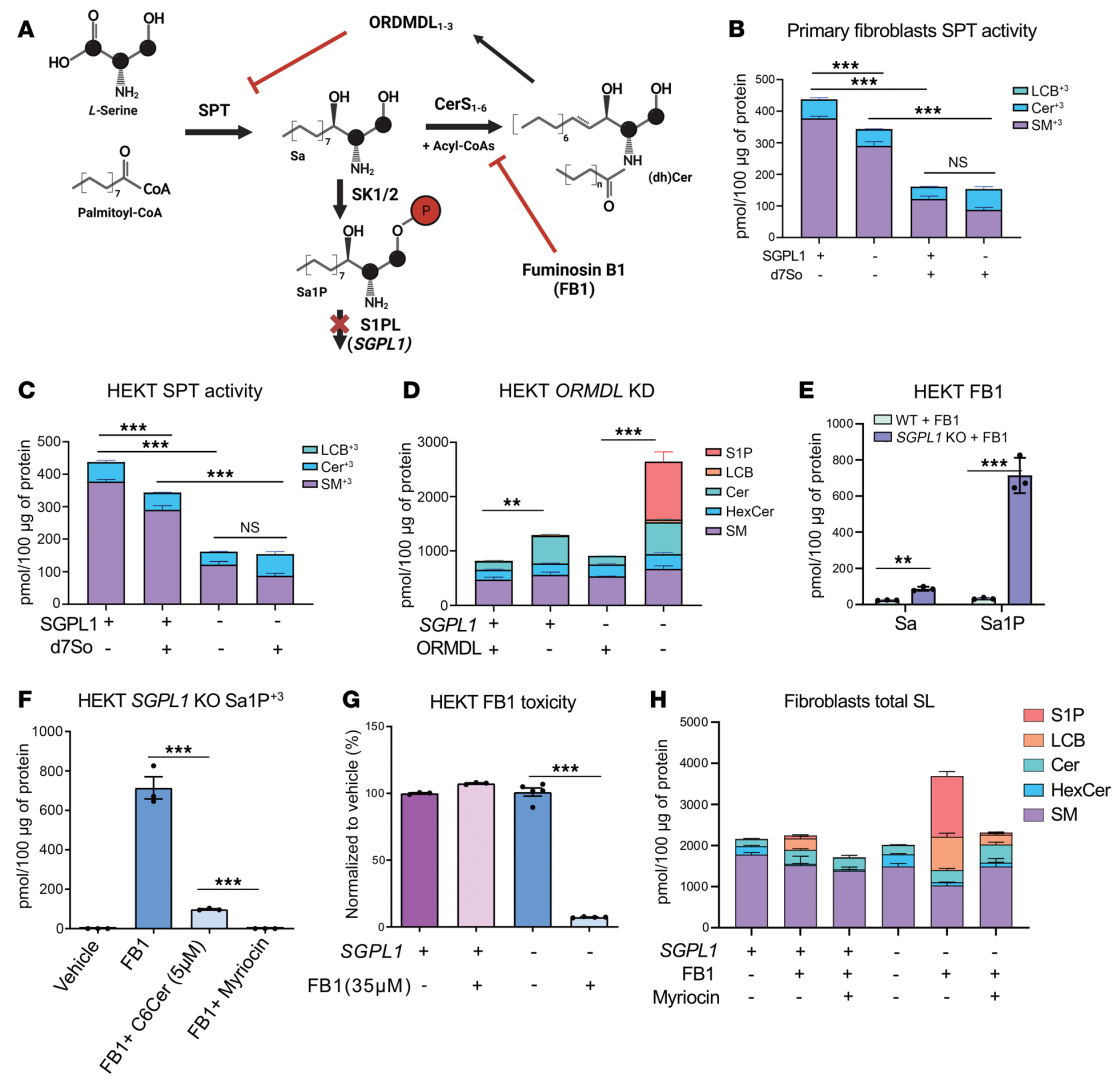
SPT regulation via the ORMDL/Cer axis prevents toxic S1P accumulation in SGPL1-deficient cells. (**A**) Schematic overview of de novo SL synthesis and its regulation by SPT. (**B**) Reduced SPT activity in SPLIS primary skin fibroblasts lacking SGPL1 (SGPL1^–^) compared with control fibroblasts (SGPL1^+^). Cells were supplemented with exogenous SLs (+) or vehicle (–) to assess homeostatic regulation. (**C**) SPT activity in HEK293T SGPL1 KO cells compared with WT cells, with or without d7-So supplementation. (**D**) Total SL levels after ORMDL1–3 knockdown (KD) in HEK293T SGPL1 KO and WT cells. Cells were transfected with scrambled (ORMDL^+^) or ORMDL-targeting siRNAs (ORMDL^–^) using Lipofectamine 3000 for 72 hours before analysis. (**E**) Total Sa and sphinganine-1-phosphate (Sa1P) levels in HEK293T SGPL1 KO and WT cells treated with the Cer synthase inhibitor FB1. (**F**) De novo formation of Sa1P in SGPL1 KO cells treated with vehicle, FB1, FB1 plus cell-permeable C6Cer, or FB1 plus the SPT inhibitor myriocin. (**G**) FB1 toxicity assay in HEK293T SGPL1 KO and WT cells. Cells were treated with FB1 for 72 hours and ATP levels were quantified using the CellTiter-Glo assay. (**H**) Total SL levels in SPLIS fibroblasts (SGPL1^–^) and control fibroblasts (SGPL1^+^) treated with vehicle (MeOH), FB1, or FB1+myriocin. SPT activity was determined by measuring incorporation of d_3_-^15^N-serine after 24 hours. Plots represent mean ± SD (*n* = 3). HexCers represent the sum of glucosylceramide and galactosylceramide. SL levels were quantified by LC-MS/MS after lipid extraction. Statistical significance was assessed using Student’s *t* test with multiple-testing correction using the 2-stage step-up method of Benjamini, Krieger, and Yekutieli. **P < 0.01; ***P < 0.001. Toxicity assay data were normalized to the mean of vehicle-treated cells (*n* = 4). Created in BioRender.

SPT activity and de novo SL synthesis are regulated by ORMDL proteins (ORMDL1, ORMDL2, and ORMDL3) in response to intracellular Cer levels (Figure 2A) (15). To see whether SL de novo synthesis was altered in SPL/SPL-deficient cells, we performed a stable isotope-labeling assay using d_3_-^15^N-serine. Primary SPLIS fibroblasts as well as HEK293T *SGPL1* KO cells had decreased SL de novo synthesis compared with controls. Supplementing cells with exogenous d7-sphingosine (d7-So) further reduced de novo SL synthesis in both KO and WT cells, indicating that an increase in intracellular SL levels leads to an enhanced compensatory downregulation of de novo synthesis (Figure 2, B and C). After silencing ORMDL1–3 expression by siRNA, we observed an increased de novo formation and a substantial accumulation of Sa1P and S1P (shown as sum of both in Figure 2D) in SPL/SPL-deficient cells (Figure 2D and Supplemental Figure 2).

In a second approach, we modulated the homeostatic control by adding fumonisin B1 (FB1), a pan-inhibitor of Cer synthases 1–6 that prevents the de novo formation of dihydroceramide and, subsequently, Cer downstream of SPT (Figure 2A). FB1 leads to a substantial accumulation of de novo–formed Sa and Sa1P in *SGPL1* KO cells but not in WT cells (Figure 2E). This accumulation was significantly reduced when activating the inhibitory SPT subunits ORMDL1–3 with cell-permeable short-chain C6-Cer or by inhibiting SPT with myriocin (Figure 2F). In comparison, FB1 was significantly more toxic for *SGPL1* deficient cells compared with WT cells (Figure 2G). In the presence of FB1, levels of phosphorylated and nonphosphorylated long-chain bases (LCBs) increased predominantly in SPL-deficient cells (Figure 2H).

### Synthesis of higher SL is an “escape” mechanism for preventing toxic S1P accumulation in SPLIS.

**Figure 3:**
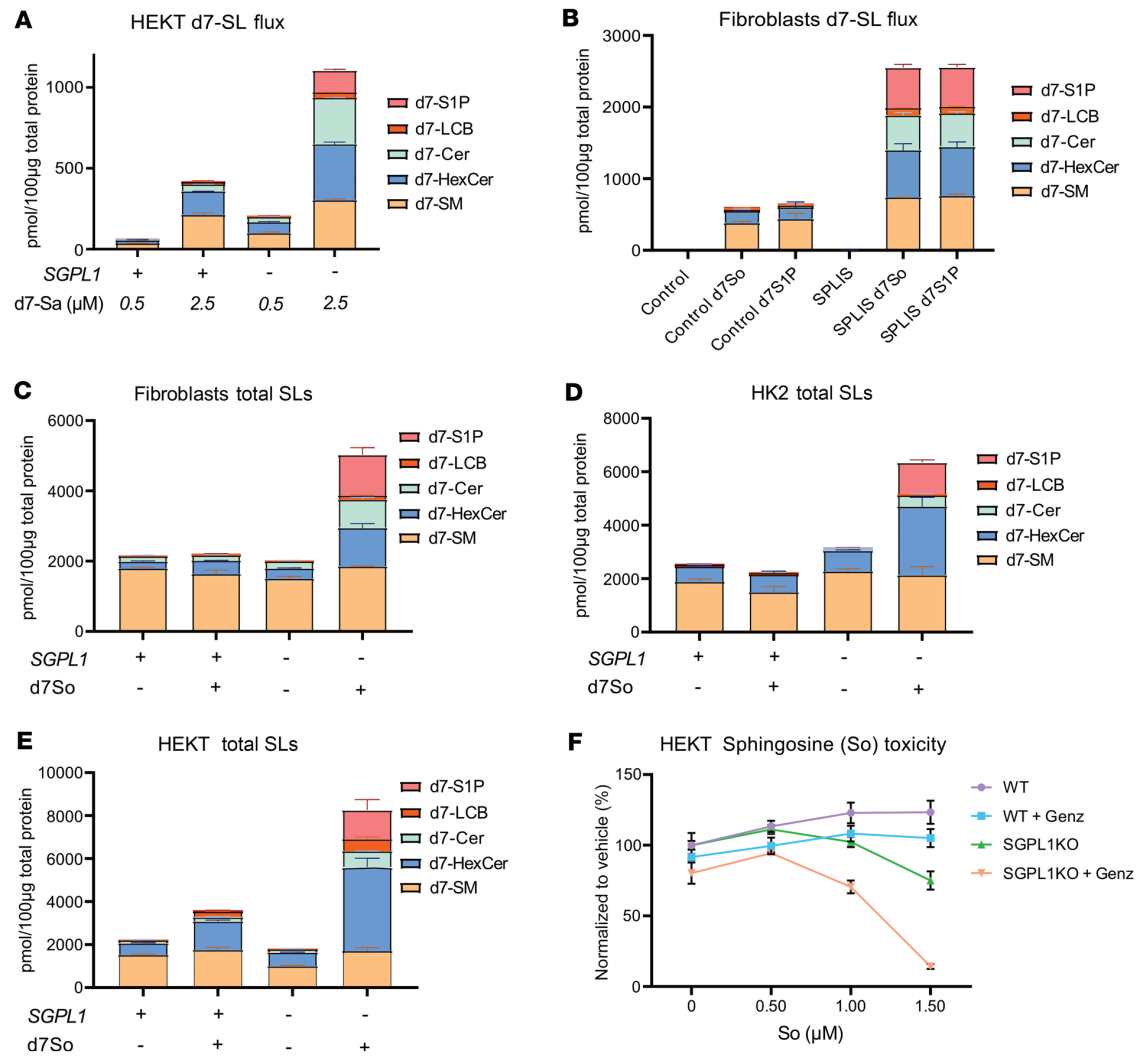
Synthesis of higher-order SLs acts as an “escape” mechanism to prevent toxic S1P accumulation in SPLIS. (**A**) d7-SL profiles following supplementation with d7-Sa (0.5 μM or 2.5 μM) in *HEK293T* *SGPL1* KO and WT cells. (**B**) d7-SL levels in primary SPLIS fibroblasts and control fibroblasts after treatment with vehicle, d7-So (0.5 μM), or d7-S1P (0.5 μM) for 24 hours. (**C**–**E**) Total SL levels in 3 different *SGPL1*-deficient cell lines — primary fibroblasts (**C**), HK2 cells (**D**), and *HEK293T* cells (**E**) — compared with corresponding controls after 24-hour supplementation with vehicle (MeOH) or d7-So (2.0 μM). Total SL levels were calculated as the sum of d7-labeled and unlabeled SL species. Bar and stacked plots represent mean ± SD (*n* = 3) for the indicated SL classes. Galactosylceramides and glucosylceramides are cumulatively represented as HexCers. SL levels were quantified via LC-MS/MS following lipid extraction. (**F**) ATP-based So toxicity assay in *HEK293T* *SGPL1* KO and WT cells. Glucosylceramide synthesis was inhibited using the GCS inhibitor Genz-123346 (Genz). Cells were exposed to increasing concentrations of So for 72 hours, and total ATP levels were quantified using the CellTiter-Glo assay. Data from the toxicity assay were normalized to the average of vehicle-treated cells (*n* = 4).

To circumvent SPT regulation by the ORMDLs, we added isotope-labeled d7-sphinganine (d7-Sa) directly to the cells and monitored the isotopic flux. The downstream products formed, such as d7-Cer, d7-HexCer, and d7-SM, showed a 3- to 4-fold increase (Figure 3A). Additionally, supplementation with d7-So or d7-sphingosine-1-phosphate (d7-S1P) at concentrations of 0.5 μM or 2.5 μM resulted in a significant increase in d7-Cer, d7-HexCer, d7-SM, and d7-S1P levels (Figure 3B). Total SL levels were calculated as the sum of all nonlabeled and isotope labelled (M+7) SL species. Overall, SPLIS primary fibroblasts, *HEK293T* *SGPL1* KO cells, and human kidney proximal tubule cell line (HK2) *SGPL1* KO cells had elevated SL levels compared with WT cells. Notably, in addition to S1P, levels of Cer and HexCer were predominantly increased, whereas total SM levels were minimally changed (Figure 3, D and E). Total S1P levels were elevated in all 3 KO cell lines. However, all KO cell lines accumulated substantial amounts of HexCer (depicted in blue in Figure 3, C–E).

To assess whether the conversion of excess SLs to HexCer is crucial for maintaining SL homeostasis and avoiding toxic SL accumulation in SPLIS, we incubated WT and *SGPL1* KO cells with increasing concentrations of So in the presence of a glucosylceramide synthase (GCS) inhibitor (Genz-123346). Blocking GCS indeed sensitized *SGPL1* KO cells to So toxicity, indicating that the conversion of excess SLs into HexCer serves as a metabolic escape mechanism to prevent toxic S1P accumulation (Figure 3F).

### SPLIS-inducing variants of SGPL1 diminished the cellular ability to clear excess S1Ps.

**Figure 4:**
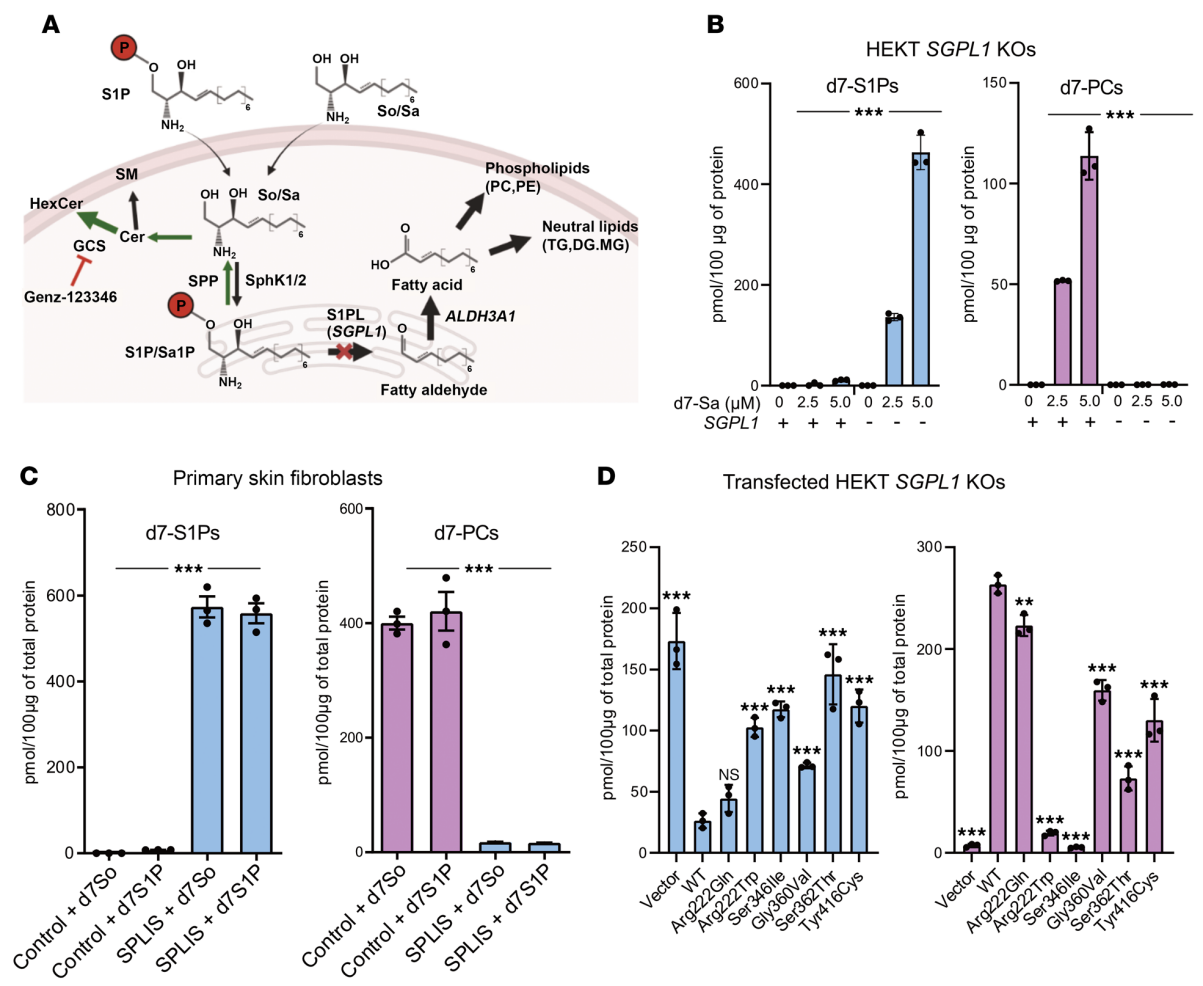
Analysis of SL metabolism and the impact of *SGPL1* mutations. (**A**) Schematic representation of the metabolism of externally supplemented SLs. (**B**) Levels of d7-S1Ps and d7-PCs after 24 hours of incubation with increasing concentrations of d7-Sa in *HEK293T* *SGPL1* KO (*SGPL1*^–^) and WT (*SGPL1*^+^) cells. Increasing d7-Sa concentrations correlate with higher d7-PC levels in WT cells, a capability diminished in *SGPL1* KO cells, which instead show an accumulation of d7-S1P. (**C**) Levels of d7-S1P and d7-PC after 24 hours of incubation with vehicle (d7-So, 0.5 μM), or d7-S1P (0.5 μM) in SPLIS fibroblasts compared with control fibroblasts. (**D**) Levels of d7-S1P and d7-PC after 24 hours of incubation with d7-Sa (2.0 μM) in *HEK293T* *SGPL1* KO cells expressing WT *SGPL1*, an empty vector, or 6 SPLIS-associated *SGPL1* variants. Bar plots represent mean ± SD (*n* = 3). d7-S1P and d7-PC levels were analyzed using LC-MS/MS following lipid extraction. Statistical significance was analyzed by 1-way ANOVA followed by Dunnett’s multiple-comparison test comparing each mutant with WT. **P < 0.005, ***P < 0.0005. Created in BioRender.

SPL converts S1P into hexadecenal and phosphoethanolamine. Hexadecenal is further oxidized by aldehyde dehydrogenase 3A2 into a fatty acid, which is subsequently metabolized into other lipids, including glycerophospholipids such as phosphatidylcholine (PC) (Figure 4A) (16). Increasing concentrations of supplemented d7-Sa correlated with the formation of d7-labeled PCs in WT cells. This conversion was absent in *SGPL1* KO cells, which instead showed a marked accumulation of d7-S1P (Figure 4B). Additionally, other d7-labeled downstream phospholipids, such as d7-phosphatidylethanolamines (d7-PEs), d7-triglycerides (d7-TGs), and d7-diacylglycerols (d7-DGs), were detected (data not shown). Similarly, when primary SPLIS fibroblasts were supplemented with either d7-So or d7-S1P, WT cells efficiently converted the labeled SL into d7-PC. In contrast, SPLIS fibroblasts primarily converted the labeled SL into d7-S1P, with minimal formation of d7-PC (Figure 4C).

Different SPLIS mutations are associated with varying disease severities. To investigate this, we compared the activity of 6 *SGPL1* variants by expressing them in *HEK293T* *SGPL1* KO cells (Figure 4D) and analyzing their ability to convert d7-Sa into d7-PC. All disease-associated variants demonstrated a reduced capacity to degrade d7-S1P and to form d7-PC. Notably, the most prevalent SPLIS variant, p.Arg222Gln (6), exhibited the highest residual activity (approximately 10%) and the highest protein expression (Supplemental Figure 2B and Supplemental Figure 3).

### Sgpl1 KO mice accumulate large quantities of S1P in kidneys and urine.

SPL deficiency results in an inability to efficiently clear exogenously supplemented SLs. Intracellularly, S1P levels are typically maintained in the nanomolar range, whereas S1P is highly abundant in plasma (~0.5 μM). Because the kidneys process large volumes of blood, they are consistently exposed to high levels of extracellular S1P. To prevent toxic intracellular accumulation of S1P, kidney cells must actively degrade this metabolite.

**Figure 5:**
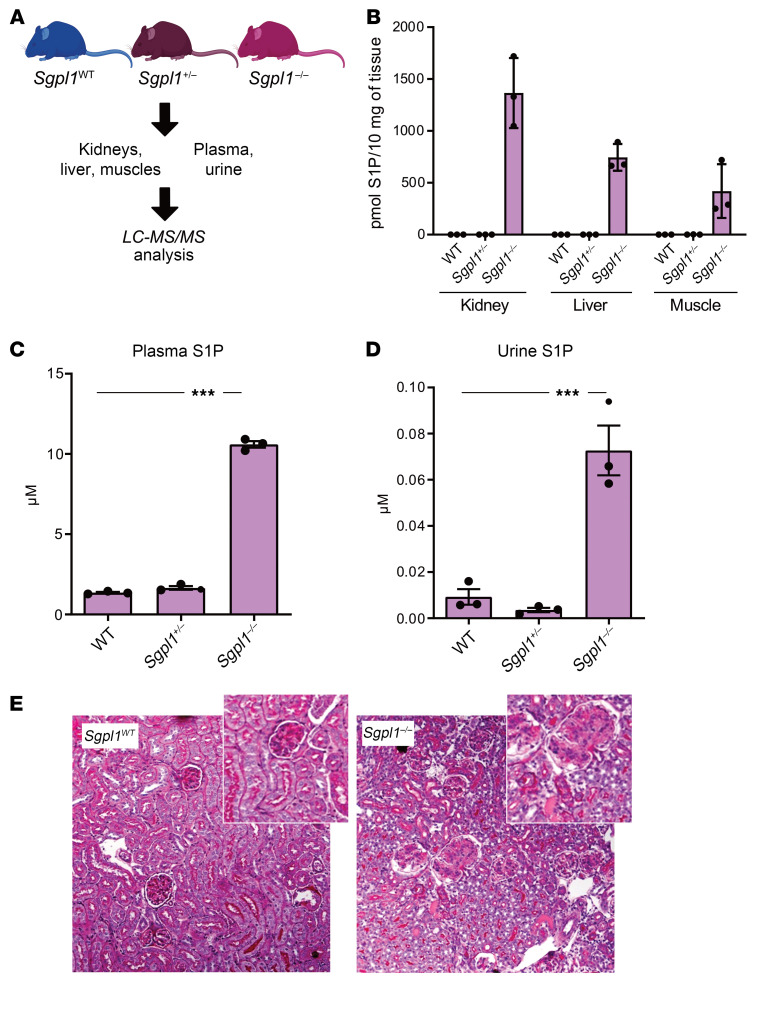
Analysis of S1P levels and kidney pathology in *Sgpl1* mice. (**A**) Schematic representation of tissue and body fluid sample collection from *Sgpl1* WT, *Sgpl1^+/–^*, and *Sgpl1^–/–^* mice. (**B**) Total S1P levels in tissues from *Sgpl1* WT (*n* = 3), *Sgpl1^+/–^* (*n* = 3), and *Sgpl1^–/–^* (*n* = 3) mice. (**C**) Total plasma S1P levels in *Sgpl1* WT (*n* = 3), *Sgpl1^+/–^* (*n* = 3), and *Sgpl1^–/–^* (*n* = 3) mice. (**D**) Total urinary S1P levels in *Sgpl1* WT (*n* = 3), *Sgpl1^+/–^* (*n* = 3), and *Sgpl1^–/–^* (*n* = 3) mice. Bar plots represent mean ± SD (*n* = 3). S1P levels were measured using LC-MS/MS. Statistical significance was calculated using a 2-tailed *t* test. Differences between WT and *Sgpl1^–/–^* were highly significant (*P* < 0.0001). (**E**) Kidney cortex histology of WT and *SGPL1* KO mice stained with PAS stain. (Left, with inset detail) WT kidney cortex shows uniform glomeruli with normal size and cellularity. (Right, with inset detail) *SGPL1* KO kidney cortex displays protein casts and glomeruli with heterogeneity in size and appearance, mesangial expansion, hypercellularity, and collagen deposition. Percent glomerulosclerosis: WT = 0%; KO = 37%; 50 or more glomeruli were analyzed per genotype. Scale bar: 100 μm. Created in BioRender.

*Sgpl1^–/–^* mice develop nephrosis characterized by hypoalbuminemia and an elevated urine albumin/creatinine ratio, mirroring the pathology observed in humans. We analyzed total S1P content in the kidney, liver, and muscle of WT, *Sgpl1^+/–^*, and *Sgpl1^–/–^* mice. Compared with WT animals, *Sgpl1^–/–^* mice had increased S1P levels in tissues and urine (Figure 5, A–D). The most pronounced S1P accumulation was observed in the kidneys of *Sgpl1^–/–^* mice, which is the primary organ affected in SPLIS. Glomerulosclerosis in these mice was confirmed histologically (Figure 5E). In contrast, heterozygous *Sgpl1^+/–^* mice did not exhibit significant S1P accumulation, suggesting that a single functional allele is sufficient to maintain normal S1P levels.

The uptake of extracellular sphingoid bases is toxic to *Sgpl1*-KO cells (Figure 3F). We hypothesize that a similar mechanism underlies the specific nephrotoxic effects seen in SPLIS. Kidney cells of *Sgpl1^–/–^* mice absorb S1P from plasma and urine but are unable to degrade the intracellular excess of SL. The kidney contains many highly specialized cell types, some of which may be particularly vulnerable to the toxic effects of S1P due to their exposure to both excreted and reabsorbed lipids in patients with SPLIS.

### Intracellular accumulation of S1P causes transient cell contraction in SPLIS fibroblasts.

**Figure 6:**
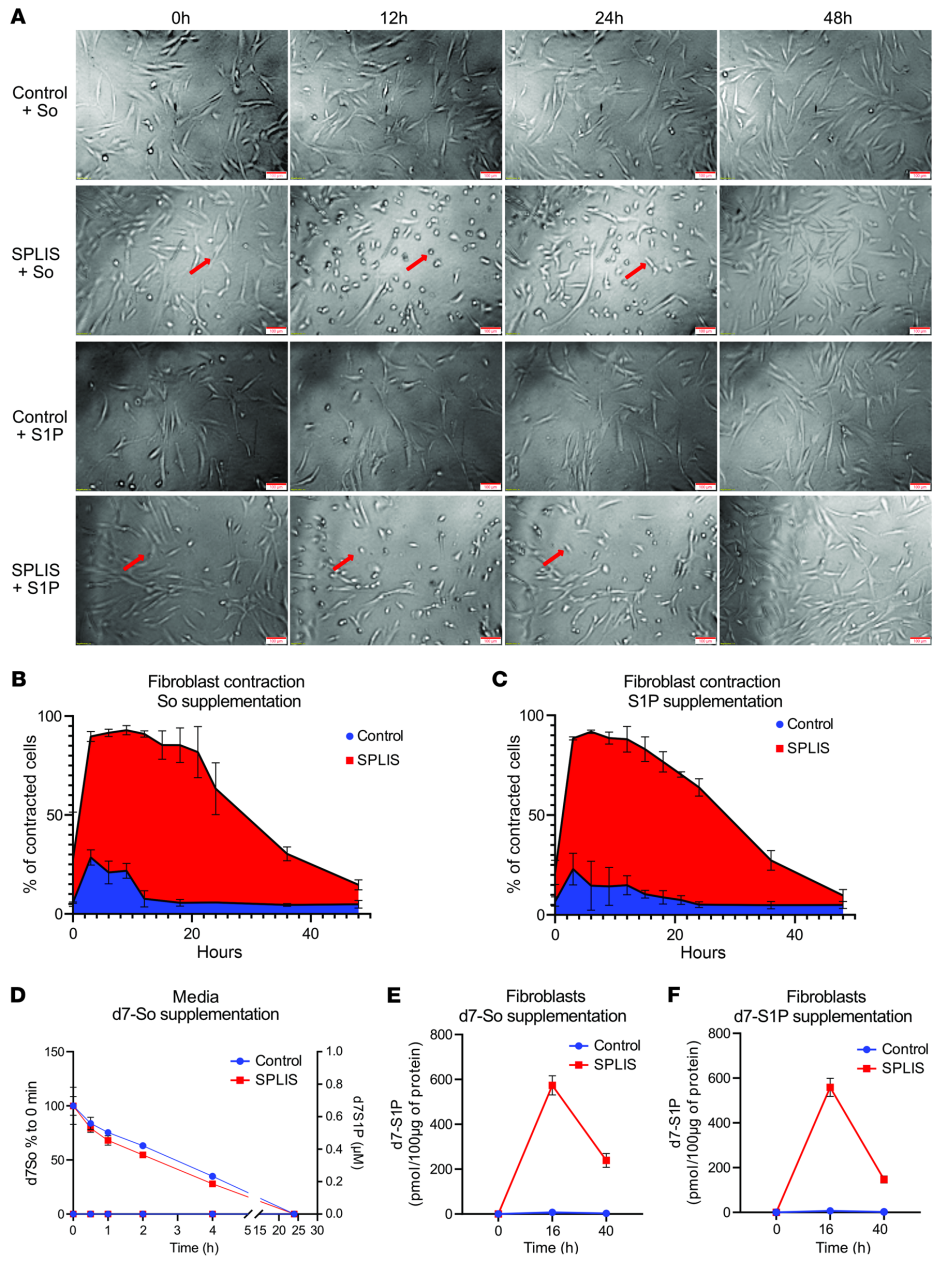
Cytoskeletal dynamics and lipid metabolism in SPLIS fibroblasts after So or S1P supplementation. (**A**) Live-cell imaging of SPLIS fibroblasts showing cell contraction after So or S1P supplementation. Red arrows indicate contracted cells. Scale bars: 100 μm. (**B** and **C**) Quantification of the percentage of contracted cells per well after So or S1P supplementation in SPLIS fibroblasts and control fibroblasts. (**D**) Time-dependent uptake of d7-So (left axis) and release of d7-S1P (right axis) into the medium in cultured SPLIS and control fibroblasts. Data were normalized to the 0-hour time point. d7-So was fully absorbed within 24 hours, with no parallel release of d7-S1P into the medium. (**E**) Timeline of intracellular d7-S1P levels after supplementation with d7-So (0.5 μM) in SPLIS and control fibroblasts. (**F**) Timeline of cellular d7-S1P levels after supplementation with d7-S1P (0.5 μM) in SPLIS and control fibroblasts. Data points are represented as mean ± SD (*n* = 3). SL levels were analyzed using LC-MS/MS after lipid extraction.

Exogenous supplementation with So or S1P leads to intracellular S1P accumulation in SPL-deficient cells (Figure 3). This increase in intracellular S1P is associated with a transient cell-rounding phenotype in SPLIS fibroblasts. Approximately 6–12 hours after the addition of So or S1P, the cells contract and subsequently reflatten after 24 hours. This behavior was not observed in control fibroblasts (Figure 6, A–C).

We compared the kinetics of So and S1P absorption from the medium and the resulting intracellular S1P accumulation. Cells were incubated with d7-So or d7-S1P for 0, 16, and 40 hours. Both lipids were effectively absorbed and cleared from the medium within 24 hours at similar rates (Figure 6D). We did not detect d7-S1P release into the medium, indicating that the absorbed lipids were primarily metabolized intracellularly. In d7-So–treated SPLIS fibroblasts, intracellular d7-S1P levels increased transiently during the first 16 hours and returned to baseline by 40 hours (Figure 6E). This transient increase in intracellular S1P was closely associated with the rounding phenotype in SPLIS cells, but not in WT fibroblasts. A similar kinetic profile was observed when supplementing d7-S1P (Figure 6F).

These findings suggest the transient rise in intracellular S1P influences the cytoskeletal dynamics of the cells, which may be relevant to the pathogenesis of SPLIS. In fact, supplementation with S1P or So in a cell migration assay confirmed the transient contraction phenotype, with reflattening after 24 hours coinciding with the initiation of cell migration. Control fibroblasts did not show this behavior (Figure 6A).

The addition of FB1 caused an increase in Sa1P levels in SPLIS fibroblasts (Figure 2E), leading to progressive cell rounding and eventual cell death, whereas control fibroblasts remained unaffected. This FB1-induced phenotype was rescued by cotreatment with the SPT inhibitor myriocin, supporting the notion that the toxic response to FB1 is a consequence of de novo SL synthesis (Figure 6B).

### Inhibiting ROCK prevents cytoskeletal changes in SPLIS fibroblasts and a SGPL1-deficient HK2 cell line.

**Figure 7:**
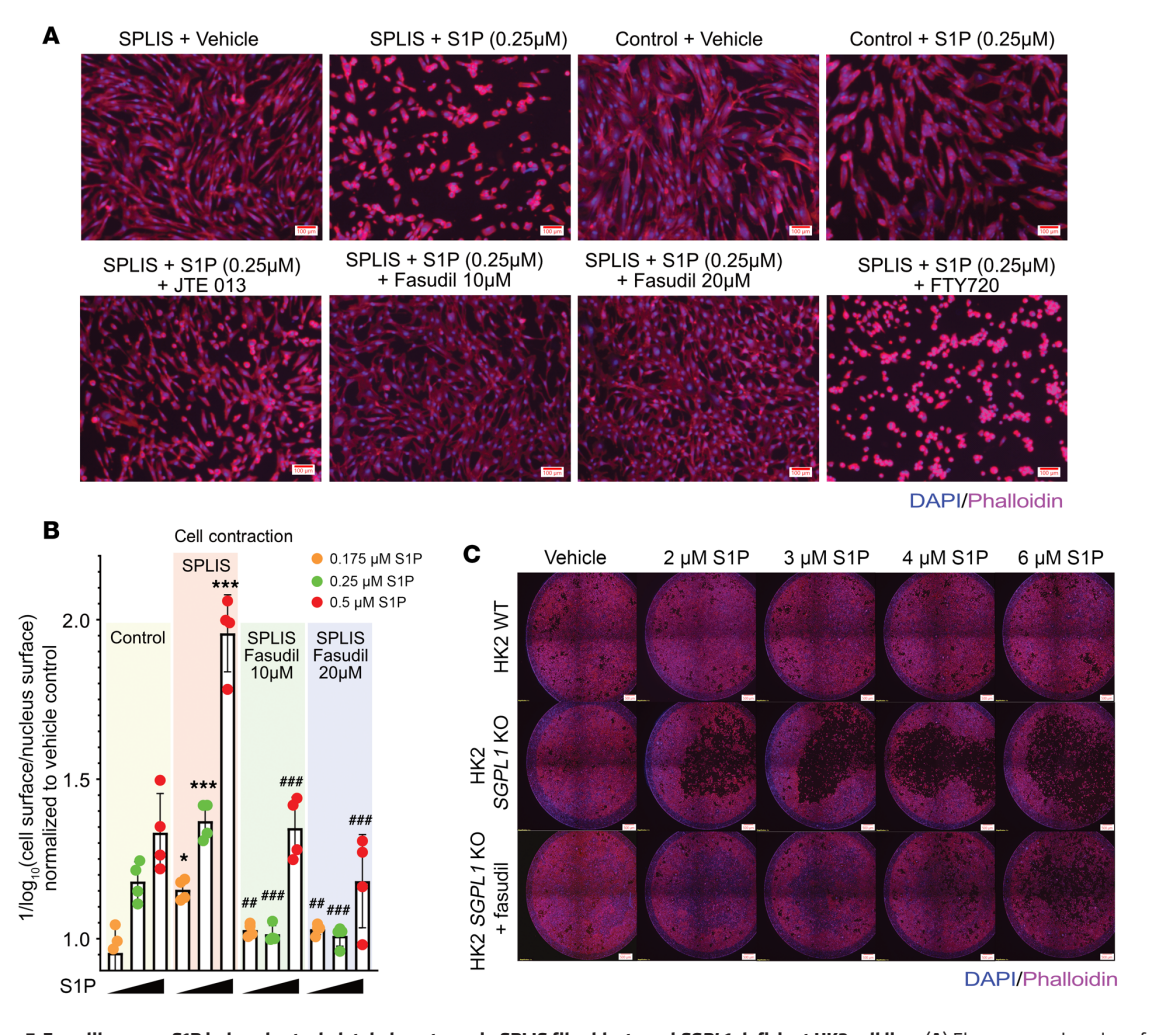
Fasudil rescues S1P induced cytoskeletal phenotypes in SPLIS fibroblasts and *SGPL1*-deficient HK2 cell line. (**A**) Fluorescence imaging of SPLIS primary fibroblasts and control fibroblasts treated with vehicle (MeOH) or S1P (0.25 μM) for 6 hours. Cells were also treated with the ROCK inhibitor (fasudil), an S1P receptor 2 inhibitor (JTE013), or an S1P receptor 1 modulator (fingolimod; FTY720). After treatment, cells were fixed with 4% paraformaldehyde (PFA) and stained with phalloidin (actin) and DAPI (nucleus). Scale bars: 100 μm. (**B**) Quantification of cell contraction. Compiled images of whole wells were analyzed using CellProfiler software. Cell contraction was defined by the formula 1/log_10_(cell surface/nucleus surface). Values were normalized to the average of vehicle-treated cells. Data are represented as mean ± SD (*n* = 4). Statistical analysis was performed using a 2-tailed parametric *t* test. Comparison between control (yellow bar) and SPLIS fibroblasts at the same S1P concentration (orange bar) (*P < 0.05, ***P < 0.0005). Comparison between SPLIS fibroblasts treated without or with 2 concentrations of fasudil (ROCK inhibitor) (green bar and blue bar) (^##^*P* < 0.005, ^###^*P* < 0.0005). (**C**) Impairment of renal epithelium formation in *SGPL1* KO HK2 cells after S1P supplementation. *SGPL1* KO or WT HK2 cells were grown for 72 hours in the presence of increasing S1P concentrations, with or without fasudil, as indicated. Cells were fixed with 4% PFA and stained with phalloidin (actin) and DAPI (nucleus). Whole wells were imaged using fluorescence microscopy. Representative images from 3 independent replicates are shown. Scale bars: 500 μm.

S1P signals through 5 G-protein–coupled S1P receptors (S1PR1–5), with S1PR2 playing a crucial role in cytoskeletal regulation. Upon S1P binding, S1PR2 activates the Rho-associated coiled-coil-containing protein kinase (ROCK) pathway, which promotes actin stress fiber assembly and focal adhesion formation. These processes are essential for maintaining cell shape, motility, and stability. To determine whether the observed cytoskeletal effects of S1P in SPLIS fibroblasts are linked to this pathway, we inhibited ROCK using the specific inhibitor fasudil. Additionally, we compared the effects of fingolimod (FTY720), an antagonist of S1PR1, 3, 4, and 5 (but not S1PR2), and JTE013, a specific S1PR2 antagonist (17). The contraction phenotype was assessed in an *SGPL1*-deficient HK2 using phalloidin staining (Figure 7). Inhibiting the Rho/ROCK axis with fasudil prevented the contraction phenotype in SPLIS cells. JTE013 showed a partial rescue effect, whereas FTY720 had no effect, indicating that S1PR2 is responsible for the contraction phenotype.

For quantitative analysis, we used the software tool CellProfiler. Cell contraction was quantified per cell and defined by the function 1/log_10_(cell surface/nucleus surface). Averages from 4 images per well were used for the analysis. SPLIS fibroblasts exhibited significantly increased cell rounding in response to S1P treatment, which was reduced in the presence of fasudil (Figure 7B). Finally, we tested the effect of fasudil on HK2 kidney cells. *SGPL1* KO or WT HK2 cells were cultured with increasing concentrations of S1P in the presence or absence of fasudil. In *SGPL1* KO HK2 cells, S1P disrupted the formation of a coherent epithelial layer, a defect that was reversed when fasudil was co-administered (Figure 7C). In summary, these findings suggest that inhibition of the Rho/ROCK signaling axis can rescue SPLIS-induced cytoskeletal phenotypes.

### Fasudil ameliorates renal dysfunction and structural damage in SPL-deficient mice.

**Figure 8:**
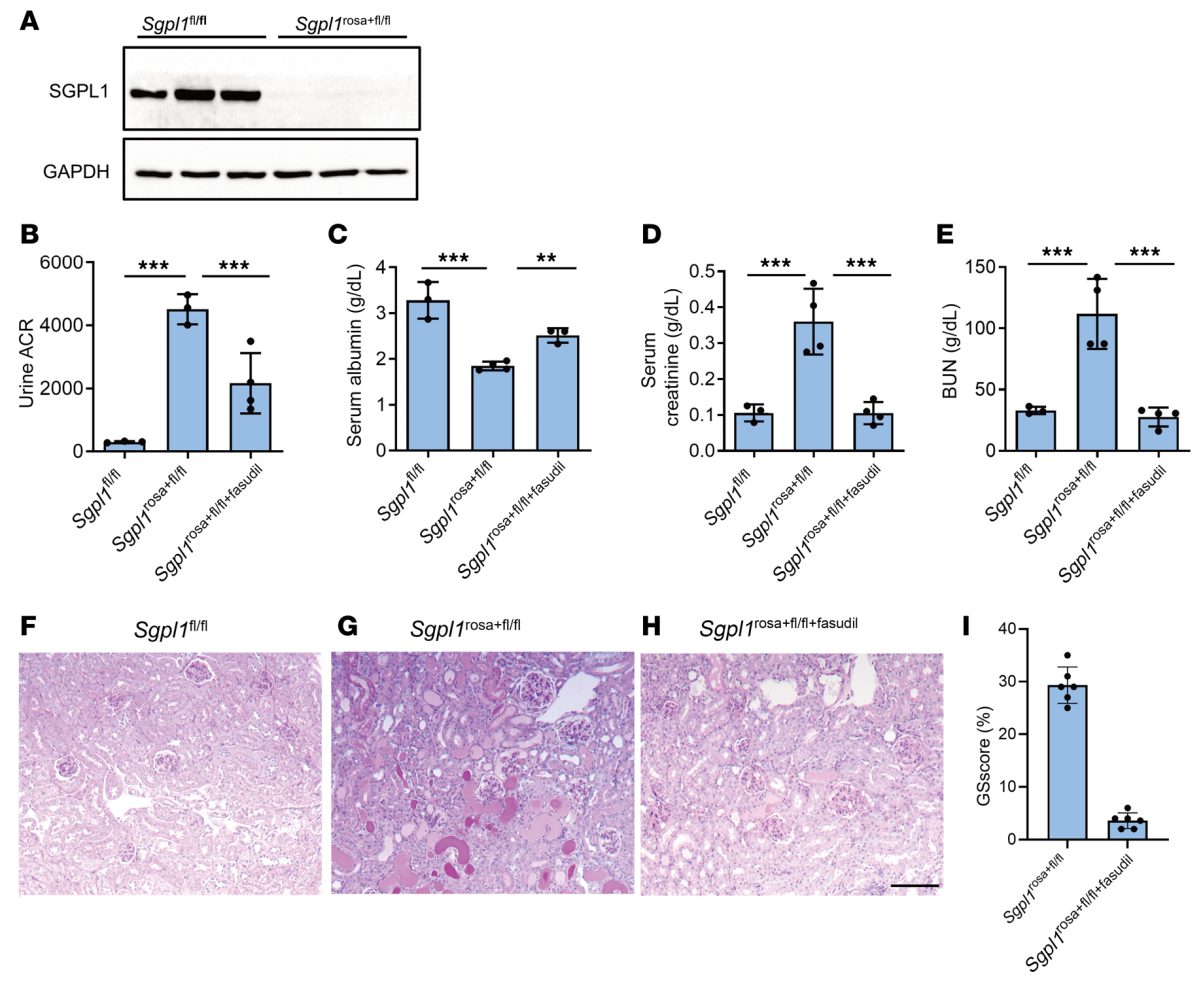
Effect of fasudil treatment in *Sgpl1^rosa+fl/fl^* mice. (**A**) Immunoblotting from the kidneys of *Sgpl1^fl/fl^* and *Sgpl1^rosa+fl/fl^* mice confirms the successful disruption of SPL in *Sgpl1^rosa+fl/fl^* mice after tamoxifen induction. (**B**–**E**) Urine ACR (μg of albumin/mg of creatinine), Serum albumin, creatinine, and BUN levels in *Sgpl1^fl/fl^* (*n* = 3), *Sgpl1^rosa+fl/fl^* (*n* = 4), and *Sgpl1^rosa+fl/fl+fasudil^* (*n* = 4) mice. (**F**–**H**) PAS staining *Sgpl1^fl/fl^*, *Sgpl1^rosa+fl/fl^*, and *Sgpl1^rosa+fl/fl+fasudil^* mice. Scale bar: 100 μm. A more severe phenotype was observed only in *Sgpl1^rosa+fl/fl^* kidneys. (**I**) Glomerulosclerosis (GSscore, %) in mice treated with *Sgpl1^rosa+fl/fl^* and mice treated with *Sgpl1^rosa+fl/fl+fasudil^* (control and fasudil, respectively) was determined by visual inspection of ≥ 50 glomeruli in both kidneys of 3 mice. **P ≤ 0.05, ***P ≤ 0.01.

To test whether inhibition of the Rho/ROCK pathway can also improve renal pathology in vivo, we treated inducible *Sgpl1*-deficient mice (*Sgpl1^rosa+fl/fl^*) with the ROCK inhibitor fasudil. Successful deletion of *SGPL1* in kidney tissue after tamoxifen induction was confirmed by immunoblotting (Figure 8A). As expected, untreated *Sgpl1^rosa+fl/fl^* mice developed a pronounced nephrotic phenotype, characterized by a strong increase in the urinary albumin/creatinine ratio (ACR), reduced serum albumin levels, and elevated serum creatinine and blood urea nitrogen (BUN), indicating impaired glomerular filtration and renal failure (Figure 8, B–E).

Importantly, fasudil treatment significantly improved all major functional parameters of nephrosis in *Sgpl1^rosa+fl/fl^* mice. Urinary ACR was markedly reduced in fasudil-treated *Sgpl1^rosa+fl/fl^* mice compared with untreated KOs, indicating a substantial attenuation of glomerular barrier dysfunction (Figure 8B). In parallel, serum albumin levels were partially restored, whereas serum creatinine and BUN concentrations were significantly decreased, reflecting an improvement of global renal function (Figure 8, C–E). Histopathological analysis further supported the functional data. Periodic acid–Schiff (PAS) staining of kidney sections revealed normal pathology in *Sgpl1^fl/fl^* mice (Figure 8F) and severe glomerular damage in untreated *Sgpl1^rosa+fl/fl^* mice, including mesangial expansion, glomerular hypercellularity, and protein casts, whereas fasudil-treated *Sgpl1^rosa+fl/fl^* mice displayed a markedly improved glomerular architecture with strongly reduced structural abnormalities (Figure 8, G–H). The glomerulosclerosis was significantly reduced in the fasudil-treated mice (Figure 8I).

**Figure 9:**
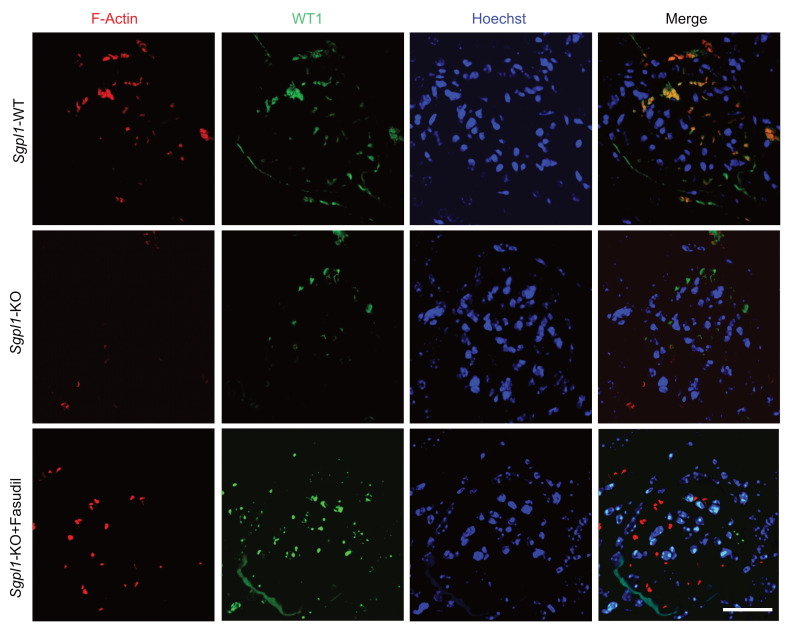
F-Actin and WT1 staining of kidneys of WT, *Sgpl1*-KO, and *Sgpl1*-KO mice treated with fasudil. Phalloidin (red), WT1 (green), and DNA (blue) showing the presence of F-actin (positive signals) in glomeruli of the WT and *Sgpl1*-KO mice treated with fasudil; a few signals of F-actin were observed in *Sgpl1*-KO glomeruli. Scale bar: 1 mm.

At the cytoskeletal level, phalloidin and WT1 co-staining demonstrated a severe loss of organized F-actin structures in glomeruli of untreated *Sgpl1^–/–^* mice. In contrast, fasudil treatment restored prominent F-actin signals within WT1-positive glomeruli, indicating recovery of podocyte cytoskeletal organization (Figure 9). Together, these data demonstrate that ROCK inhibition by fasudil significantly improves both functional and structural manifestations of nephropathy in SPL-deficient mice.

## Discussion

SPLIS, or nephrotic syndrome type 14, is an inherited disease caused by recessive mutations in *SGPL1*. Because SPL is responsible for the terminal degradation of SLs, reduced SPL activity is expected to result in increased intracellular SL levels. However, the underlying metabolic rearrangements caused by SPL insufficiency remain poorly understood. Additionally, the pathophysiological mechanisms of SPLIS leading to nephrosis are not well characterized. Here, we report the case of a patient with a novel SPLIS-associated *SGPL1* variant (p.Ser362Thr). The patient presented with congenital nephrotic syndrome and immunodeficiency, resulting in early death at 9 months of age. Contrary to previous studies (18), we did not observe significantly increased S1P levels in the patient’s plasma and found only slightly elevated intracellular S1P levels in patient-derived skin fibroblasts. Instead, we detected substantially increased levels of Cer, SM, and HexCer (Supplemental Figure 1).

However, circulating S1P levels can be suppressed during acute critical illness. Systemic inflammation such as sepsis is associated with reductions in plasma S1P (19), indicating that severe illness could mask genotype-specific effects. Because the patient carrying the S362T mutation was critically ill at the time of sampling, the measured plasma S1P concentrations may have underestimated the mutation-related metabolic disturbance. Due to the young age and critical condition of the patient, only very little plasma was available, and the lipid extraction methodology we used was developed for a general lipidomics analysis and not optimized for S1P recovery.

SL de novo formation is initiated by SPT and regulated by its subunits ORMDL1–3 in response to intracellular Cer levels (15, 20). Increasing intracellular SL content by supplementing So, Sa, or membrane-permeable C6-Cer reduced SPT activity and lowered SL de novo synthesis in both WT and SPL-deficient cells (15). In fact, SPT activity and SL de novo synthesis are markedly reduced in SPL-deficient cells, including patient-derived fibroblasts. Reduced SPT activity has also been reported in SPL-deficient neurons (21). We hypothesize that in SPLIS, the impaired breakdown of SL is partially offset by a decrease in SL de novo synthesis, thereby helping to maintain low intracellular S1P levels despite the absence of SPL activity. In contrast, inhibiting Cer synthesis with FB1 or lowering ORMDL1–3 expression, significantly increases SPT activity. Under conditions of increased SL synthesis, SPL-deficient cells accumulated significant amounts of S1P, which was not observed in cells expressing functional SPL. Conversely, S1P accumulation was prevented when C6-Cer was supplemented in addition to FB1. These findings suggest that in the absence of SPL, SPT regulation via the Cer-ORMDL axis is crucial for maintaining baseline SL levels. This hypothesis is supported by data from *Drosophila*, where the muscle phenotypes in flies with hypomorphic mutations in *Sply* (the fly ortholog of SPL) were attenuated by crossing in a hypomorphic mutant of *lace* (the ortholog of SPT) (22). The importance of controlling SPT and SL de novo synthesis in absence of SPL activity is further supported by the higher toxicity of FB1 in SPL-deficient cells (Figure 2G). Typically, we did not observe a significant increase in intracellular S1P or a notable release of S1P into the medium in the absence of SPL (Figure 6D). However, we observed a significant increase in HexCer, suggesting that the increased synthesis of glycosphingolipids may serve as a secondary compensatory mechanism to prevent intracellular accumulation of S1P and other potentially harmful SL species. This protective mechanism might explain why supplementing low amounts of d7-Sa to SPL-deficient cells increased HexCer levels without altering S1P, but higher concentrations of d7-Sa overloaded the system’s compensatory capacity, resulting in a toxic increase in S1P. A compensatory conversion of Cer into HexCer has also been reported in SPL-deficient neurons (21). SPL-deficient cells supplemented with external SL had the highest HexCer levels. However, blocking HexCer synthesis with the GCS inhibitor *Genz-123346* sensitized SPL-deficient cells to So supplementation, further supporting the role of HexCer synthesis as a compensatory mechanism.

SPL converts S1P into fatty aldehydes, which are subsequently metabolized into fatty acids and ultimately are incorporated into glycerophospholipids (e.g., PCs, PEs) and neutral lipids (e.g., TGs, DGs) (16). In *SGPL1*-deficient cells, the inability to efficiently cleave S1P results in reduced conversion into glycerophospholipids, particularly when cells are supplemented with external LCBs such as d7-So, d7-Sa, or d7-S1P (Figure 4). This impaired conversion has been linked to reduced PE formation in SPLIS, which may compromise autophagy because lipidation of LC3 — a key step in autophagosome formation — relies on adequate PE levels (23).

The SPLIS variants of SPL reduce enzyme activity (Figure 4D), which affects the conversion of externally supplemented SL into PCs (Figure 4D). The highest residual activity was observed for *SGPL1* p.Arg222Gln, which is the most frequently reported SPLIS variant and is associated with a comparatively mild phenotype (6, 7).

We observed significant accumulation of S1P in the tissues, blood, and urine of *Sgpl1^–/–^* mice. The most pronounced S1P accumulation was in the kidneys, the primary organ affected in SPLIS. Kidneys are constantly exposed to relatively high S1P concentrations in the blood (approximately 0.5 μM), creating persistent pressure to degrade the reabsorbed S1P. Elevated S1P levels in the urine of *Sgpl1^–/–^* mice suggest that in SPLIS, tubular kidney cells exposed to high extracellular S1P levels, but incapable of degrading it, may be particularly vulnerable to its toxic effects. Given that S1P reabsorption from urine is specific to the kidneys, this inability to degrade S1P likely explains the selective nephrotic phenotype observed in SPLIS.

The supplementation of external SLs (So or S1P) to SPLIS primary fibroblasts caused profound cytoskeletal changes, manifesting as a time-dependent but transient cell contraction closely correlated with changes in intracellular S1P levels. Similarly, supplementation of S1P induced epithelial defects in SPL-deficient HK2 cells. S1P is a pharmacologically potent molecule that exerts its effects through 5 specific receptors (S1PR1–5) (24). S1PR2 links to cytoskeletal regulation via the Rho/ROCK signaling pathway (25), and altered S1PR2 signaling has been linked to kidney disease previously (26). Blocking S1PR2 with JTE013 and inhibiting the ROCK signaling axis with fasudil prevented S1P-induced cytoskeletal changes in SPLIS primary fibroblasts and *HK2* *SGPL1* KO cells.

Our in vivo data now provide direct functional and structural evidence that pharmacological inhibition of the Rho/ROCK signaling axis can significantly attenuate nephropathy in *SGPL1* deficiency. Treatment of inducible *Sgpl1*-deficient mice with the ROCK inhibitor fasudil resulted in a marked improvement of classical nephrotic parameters, including a substantial reduction of albuminuria, partial normalization of serum albumin levels, and significant decreases in serum creatinine and BUN levels. These findings demonstrate that ROCK inhibition improves glomerular barrier function and global renal performance in the context of *SGPL1* deficiency.

Histopathological analysis further corroborated the functional rescue. Untreated *Sgpl1*-deficient mice exhibited severe glomerular pathology characterized by mesangial expansion, hypercellularity, and protein casts, consistent with progressive glomerulosclerosis. In contrast, fasudil-treated animals displayed preserved glomerular architecture with a strong reduction of these pathological features. At the cellular level, phalloidin and WT1 co-staining revealed that the profound loss of organized F-actin structures in podocytes of untreated *Sgpl1*-deficient mice was largely reversed by fasudil treatment, indicating restoration of podocyte cytoskeletal integrity.

These in vivo findings strongly support our cellular data and the model in which pathological intracellular accumulation of S1P in *SGPL1*-deficient renal cells activates S1PR2/Rho/ROCK signaling, leading to actin cytoskeletal destabilization, podocyte dysfunction, and breakdown of the glomerular filtration barrier. However, the upstream link between elevated intracellular S1P and Rho/ROCK activation in SPLIS remains unclear. One possibility is that excess S1P is exported and activates S1PR2/3, which couples to G12/13–RhoA. Alternatively, intracellular S1P may affect Rho regulators directly, without involving surface receptors. The fact that ROCK inhibition restores both cytoskeletal organization and renal function identifies this pathway as a central downstream effector of S1P toxicity in SPLIS. This is fully consistent with previous work linking aberrant Rho–ROCK signaling to proteinuric kidney disease, podocyte effacement, and progressive glomerulosclerosis in other pathological settings.

From a translational perspective, these findings are highly relevant. Fasudil is a clinically established ROCK inhibitor that is approved in several Asian countries for the treatment of cerebral vasospasm following subarachnoid hemorrhage and has accumulated substantial human safety data from clinical use and experimental studies. Although Fasudil is currently not approved for SPLIS or any other chronic systemic indication in Europe or the United States, the existing clinical experience substantially facilitates translational evaluation of ROCK inhibition as a therapeutic strategy for SPLIS. Importantly, ROCK inhibition targets a downstream pathogenic signaling mechanism rather than the SL metabolic defect itself. This may avoid the inherent risks of metabolic substrate-reduction strategies, such as induction of alanine-driven deoxy-SL formation or unintended consequences of broadly suppressing SL biosynthesis.

However, fasudil does not correct the underlying inability to degrade S1P; therefore, it likely acts by blocking a key toxic signaling axis rather than eliminating the primary metabolic trigger. Long-term efficacy, optimal dosing regimens, and potential off-target effects in the setting of chronic SPLIS will require further investigation. In addition, it will be important to determine whether ROCK inhibition can also mitigate extrarenal manifestations of SPLIS, including neurological and immunological complications.

In conclusion, our data identify the Rho/ROCK pathway as a central effector of S1P-driven nephrotoxicity in *SGPL1* deficiency and establish pharmacological ROCK inhibition as a highly promising therapeutic strategy for SPLIS-associated nephropathy.

## Methods

### Sex as a biological variable.

The patient sample was from a female individual. The animal experiments were performed in mixed-sex groups. We did not observe any overt sex-dependent differences.

### Cell culture.

HEK293T cell lines and primary fibroblasts were grown in high-glucose-concentration DMEM (Thermo Fisher Scientific) supplemented with 10% FBS and 1% penicillin/streptomycin (P/S) in a 5% CO_2_ incubator at 37°C. Primary skin fibroblasts were isolated from patient and healthy control skin biopsy specimens. HK2 cell lines were grown in DMEM/F-12 (Thermo Fisher Scientific) supplemented with 10% FBS and 1% P/S in a 5% CO_2_ incubator at 37°C. For the fluorescence microscopy experiments, cells were grown in DMEM without phenol red and supplemented with 10% FBS and 1% P/S in a 5% CO_2_ incubator at 37°C. For the transfection and silencing experiments, cells were grown in high-glucose DMEM supplemented with 10% FBS without any addition of P/S. Cells were tested for mycoplasma contamination.

### SGPL1 KO cell lines.

The HEK293T *SGPL1* KO cell line was a gift from Per Haberkant (European Molecular Biology Laboratory Heidelberg, Heidelberg, Germany) and generated using CRISPR/Cas 9, as described by Haberkant et al. (27).

The HK2 *SGPL1* KO cell line was a gift from Robert Engel (German Cancer Research Center [DKFZ], Heidelberg, Germany) and generated using CRISPR/Cas9, as described previously (28).

### Plasmid generation.

WT *SGPL1* was cloned into the p.Lenti-V5 vector (V498–10, Thermo Fisher Scientific), and PCR-based site-directed mutagenesis was used to introduce SPLIS-associated *SGPL1* mutations, using primers listed in Table 2, following the manufacturer’s recommendation (Invitrogen). Generated plasmids were confirmed by Sanger sequencing.

### Generation of the SGPL1 point mutant cell lines.

HEK293T *SGPL1* KO cell lines were transfected with P.Lenti-V5 plasmid expressing WT *SGPL1* or *SGPL1* missense variants using Lipofectamine 3000 (L3000001, Thermo Fisher Scientific). pLenti-V5 containing β-galactosidase (V498–10, Thermo Fisher Scientific) was used as an empty vector control. Transfected cells were selected by culturing in DMEM medium (10% FBS) with Blasticidine (A1113903, Thermo Fisher Scientific; 20 μg/mL) for 4 weeks.

### Silencing of ORMDL123.

siRNAs targeting human *ORMDL1*, *2*, and *3* were used to silence (knockdown) ORMDL1, 2, and 3 expression. All siRNAs were mixed to an individual final concentration of 10 nM in reduced-serum medium (Opti-MEM; 31985062, Thermo Fisher Scientific). Transfection was performed using Lipofectamine RNAiMAX transfection reagent (13778150, Thermo Fisher Scientific) according to the manufacturer’s recommendations. The medium was replaced every 24 hours with fresh DMEM (10% FBS), and cells were grown for a total of 72 hours before the start of labeling experiments. Knockdown efficiency was determined using qRT-PCR.

### Isotopic labeling.

For the SL labeling assay, cells were plated in 6-well plates. Cells were grown for 48 hours to 70% confluence in DMEM (10% FBS and 1% P/S) (HEK293T, primary fibroblasts) or DMEM/F12 (10% FBS and 1% P/S) growth medium. At 24 hours before harvesting, the medium was replaced with l-Serine–free DMEM (C4331.0500, Genaxxon Bioscience) with 10% FBS and 1% P/S and supplemented with 1 mM d_3_-^15^N–l-serine (DNLM-6863-PK, Cambridge Isotope laboratories) with an addition of other treatments, including d7-Sa (860658), d7-So (860657), or d7-S1P (860659), from Avanti Polar Lipids; FB1 (F1147, Sigma-Aldrich); and myriocin (M1177, Sigma-Aldrich), as indicated in the figures. Cells were harvested by trypsinization, and the cell pellets washed twice with cold PBS. The pellets were frozen and stored at –20°C until extraction.

### Mice tissue S1P measurement.

Mice tissues were generated as described previously (29). Tissue homogenates were prepared using Precellys homogenizer. Briefly, tissues were weighed on ice and transferred to the Precellys 2 mL tubes with 6 beads per tube. MeOH was added to correct the concentration of the homogenates. Then, 9 rounds of homogenization at 5,500 rpm, 25 seconds each round, were performed at 4°C. Tissue homogenates were used for lipid extraction and analysis, as described next.

### Lipidomics analysis.

Lipidomics analysis was performed as described previously (20). Briefly, extraction was performed by mixing the cell pellets, plasma, urine, or tissue homogenate with extraction buffer (60 minutes, 37°C) consisting of a mixture of methanol: methyl *tert-*butyl ether/chloroform 4:3:3 (vol/vol/vol) and internal standards. After centrifugation (16,000*g*, 10 minutes), the single-phase supernatant was collected, dried under N_2_, and stored at –20°C. Before analysis, lipids were dissolved in MeOH and separated on a C30 LC column using gradient elution with (a) acetonitrile/water (6:4) with 10 mM ammonium acetate and 0.1% formic acid; and (b) isopropanol/acetonitrile (9:1) with 10 mM ammonium acetate and 0.1% formic acid at a flow rate of 260 μL/minute. Eluted lipids were analyzed on a Q-Exactive HRMS (Thermo Fisher Scientific) in positive and negative modes using heated electrospray ionization. MS2 fragmentation spectra were recorded in data-dependent acquisition mode. Peak integration was performed with TraceFinder 4.1 (Thermo Fisher Scientific). Lipids were identified by predicted mass (5 ppm resolution), retention time, and specific fragmentation patterns. Next, lipid concentrations were normalized to the internal standards (*n* = 1 per class) and total protein amount.

The following internal standards (all from Avanti Polar Lipids) were used: d5-1-Deoxymethylsphinganine (SPB17:0;O; 860476) 100 pmol/sample; C17 sphingosine-1-phosphate (SPBP17:0;O2; LM2144) 50 pmol/sample; 1-deoxydihydroceramide (Cer18:0;O/12:0; 860460P) 100 pmol/sample; 1-deoxyceramide (Cer18:1;O/12:0; 860455) 100 pmol/sample; dihydroceramide (Cer18:0;O2/12:0; 860635) 100 pmol/sample; ceramide (Cer18:1;O2/12:0; 860512) 100 pmol/sample; SM (SM18:1;O2/12:0; 860583) 100 pmol/sample; glucosylceramide (GlcCer18:1;O2/ /8:0; 860540) 100 pmol/sample; and SPLASH standard (330707) 2.5 μL/sample.

The following transitions were used for the identification of sphingolipids: Cers, [M+H]^+^ → [M+H − H_2_O]^+^, [M+H]^+^ → [M+H − H_2_O − FA]^+^,[M+H]^+^ → [M+H – 2 × H_2_O − FA]^+^; HexCers, [M+H]^+^ → [M+H − hexosyl]^+^, [M+H]^+^ → [M+H − H_2_O − FA − hexosyl]^+^, [M+H]^+^ → [M+H – 2 × H_2_O − FA − hexosyl]^+^; SMs, [M+H]^+^ → [PO_4_-choline]^+^, [M+H]^+^ → [M+H − H_2_O − FA – PO_4_-choline]^+^, [M+H]^+^ → [M+H − 2 × H_2_O − FA − PO_4_-choline]^+^; S1Ps, [M+H]^+^ → [M+H – 2 × H_2_O – FA – PO_4_]^+^, [M+H]^+^ → [M+H − H_2_O − FA – PO_4_]^+^; free long-chain bases (So and Sa), [M+H]^+^ → [M+H − H_2_O]^+^, [M+H]^+^ → [M+H – 2 × H_2_O]^+^; and PCs, [M+H]^+^ → [PO_4_-choline]^+^, [M+H]^+^ → [choline]^+^ and [M+formate]^+^ → [M+formate − PO_4_-choline − FA]^+^, where FA corresponds to fatty acyl.

### Protein determination.

For the protein content normalization, cell pellets remaining after the lipid extraction were dissolved in urea (8 M) containing 1% 2-mercaptoethanol and mixed at 800 rpm at 90°C (Thermomixer; Eppendorf) for 20 minutes. Next, protein extracts were snap frozen using dry ice, and the whole process was repeated 3 times. After centrifugation (21,000 × *g*, 10 minutes), supernatants were collected and used for further analysis. Total protein was determined using Bradford assay (Bio-Rad; 1:50 dilution) according to the manufacturer’s recommendation.

### Western blotting.

Protein isolation and Western blot were performed as described previously (20). Kidneys were harvested from *Sgpl1^fl/fl^* and *Sgpl1^rosa+fl/fl^* mice 28 days after tamoxifen induction. Samples were homogenized in radioimmune precipitation assay buffer (25 mM Tris-HCl, pH 7.6, 150 mM NaCl, 1% NP-40, 1% sodium deoxycholate, and 0.1% SDS) supplemented with protease inhibitors using a Tissuelyzer and then cleared by centrifugation. Primary antibodies used for immunodetection of V5-antigen were V5 Tag antibody (R960-25, Thermo Fisher Scientific; 1:2,000 dilution, 1 hour at room temperature) and, for calnexin immunodetection, anti-calnexin (ZRB1147, Sigma-Aldrich; 1:2,500 dilution, 1 hour at room temperature). We used HRP-conjugated anti-mouse (NC2294470, Thermo Fisher Scientific; 1:10,000, 1 hour at room temperature) and anti-rabbit (12-348, Thermo Fisher Scientific; 1:10,000, 1 hour at room temperature) IgGs as secondary antibodies.

The following antibodies were used to detect the protein level: anti-mouse SPL (SAB2109200, MilliporeSigma), rabbit anti-GAPDH (sc-25778, Santa Cruz Biotechnology) and HRP-conjugated secondary antibodies (111-035-144, goat anti–rabbit IgG [H + L], Jackson Immuno Research; sc-2020).

### Toxicity assay.

For the toxicity assay, cells were grown for 72 hours in 96-well plates with treatment (So: 860490, Avanti Polar Lipids; FB1: F1147, Sigma-Aldrich; Genz: 5.38285, Sigma-Aldrich), as indicated in the figures. Next CellTiter-Glo Luminescent Cell Viability Assay (G7570, Promega) was performed according to the manufacturer’s recommendations. Chemiluminescence signal was detected using TECAN infinite M 200 Pro reader. All conditions were corrected for the solvent concentration.

### Live-cell microscopy.

For the live-cell microscopy, cells were grown in 96-well plates in the incubator at 5% CO_2_ and 37°C. Indicated treatment (JTE 013 [catalog J4080], fasudil [catalog CDS021620], and FTY720 [catalog SML0700], all from Sigma-Aldrich) was added by media exchange. Then, the plate was immediately transferred to a live-cell imaging microscope (Olympus IX81) with a motorized stage, fitted with an incubator with preheated humidified atmosphere (Ibidi mixer), and kept at 37°C and 5% CO_2_. Phase-contrast images were acquired at ×20 magnification every 15 minutes for 48 hours. Four images per well were acquired and compiled. The percentage of contracted cells was acquired by selecting four 10 mm rectangles (regions of interest) per well and then contracted cells and noncontracted cells were counted manually using ImageJ software. The same positions of the rectangles per well were used at all time points and conditions. The percentage of contracted cells per well was calculated as average of 4 percentages in rectangles. All original images of cut rectangles used for manual counting are available.

### Scratch assay.

Scratch assay was performed as described previously (30). Briefly, fibroblasts were seeded in 12-well plates for 48 hours. Next, cell proliferation was stopped by 2-hour incubation with Mitomycin C (M4287, Sigma-Aldrich; 10 μg/mL). Then, a scratch was introduced in the middle of the well with a 20 μL pipette tip. To remove cell debris, the wells were rinsed once with PBS, and fresh DMEM (10% FBS and 1 % P/S) supplemented with the specified treatments was added. Plates were transferred to a live-cell imaging microscope (Olympus IX81) equipped with a motorized stage and fitted with an incubator featuring a preheated, humidified atmosphere (Ibidi mixer, Ibidi GmbH), which was maintained at 37°C and 5% CO_2_. Phase-contrast pictures were acquired at ×10 magnification every 30 minutes for 48 hours.

### Fluorescence microscopy.

For fluorescence microscopy, cells were grown in 96-well plates in an incubator at 5% CO_2_ at 37°C. Indicated treatments were added by exchange of the medium. After the given time (indicated in the figures), cells were washed 3 times with PBS and fixed using 4% paraformaldehyde for 30 minutes. Next, cells were washed 3 times with PBS and then incubated for 1 hour with DAPI (D9542, Sigma-Aldrich; 1 μM in PBS) and Phalloidin-665 (18846, Sigma-Aldrich; 0.5 μM in PBS). Cells were washed with PBS and imaged immediately. Images were acquired using a fluorescence microscope (Olympus IX81) with a motorized stage at ×20 magnification.

For the phalloidin staining, kidney sections were deparaffinized and rehydrated. Gentle heat retrieval was performed in citrate buffer pH 6.0 (10 mM, 95–98°C, ~10 minutes), then cooled in PBS. WT1 (Wilms tumor protein; catalog sc-7385), a marker for glomeruli, and Alexa Fluor 568 phalloidin (ThermoFischer Scientific, A12380) in normal horse serum were applied and incubated for 30 minutes at room temperature in the dark. Hoechst in VectaShield (Vector Laboratories) was used to mount the slides. Images were captured by a Zeiss LSM 710 confocal microscope.

### Animals.

*Sgpl1* KO mice in which the *Sgpl1* gene is constitutively disrupted (*Sgpl1^–/–^* mice) have been described previously (31). Heterozygous KO breeders were mated, and newborns were genotyped by toe biopsy. Transgenic mice harboring *Sgpl1^fl/fl^* and *Sgpl1^rosa (cre/ERT2)+fl/fl^* alleles were gifted from Jesus Rivera-Nieves (UCSD, La Jolla, California) and have been described previously (31).

### Studies.

Animal experiments were approved by the IRB UCSF (San Francisco). The patient sample was collected for diagnostic purposes. All participants involved provided written informed consent.

### Serum albumin, creatinine, and BUN.

Serum albumin, creatinine, and BUN levels were measured using the COBAS INTEGRA 400 plus instrument (Roche Diagnostics) by the UCD Comparative Pathology Laboratory.

### Determination of ACR.

The ACR in urine from *Sgpl1^fl/fl^*, *Sgpl1^rosa+fl/fl^*, and *Sgpl1^rosa+fl/fl+fasudil^* was determined using the Ethos/Exocell’s immunospecific albumin ELISA and creatinine companion kit according to the manufacturer’s protocol.

### PAS staining.

PAS staining was performed on 5 micron kidney sections from *Sgpl1^fl/fl^*, *Sgpl1^rosa+fl/fl^*, *Sgpl1^rosa+fl/fl+fasudil^*, *Sgpl1^–/–^*, and WT mice, as we described previously (32). The percentage of glomerulosclerosis was determined by visual inspection.

A total of 50 glomeruli and surrounding tissues were evaluated to quantify abnormalities, including segmentally sclerosed glomeruli, glomeruli displaying mesangial hypercellularity, and interstitial fibrosis. Both kidneys of 3 mice per group were analyzed. Casts were scored as either not present or as 1, 2, 3, or 4+ in severity, with 1+ indicating rare casts and 4+ indicating 80% or more tubules filled with PAS-positive material.

### Image analysis.

Image analysis was performed with CellProfiler 4.2.1 using an in-house made pipeline. First, the nucleus area was segmented as a primary object (Otsu thresholding method) using the DAPI channel image. Second, cellular area was identified and segmented as a secondary object from nuclear area in the phalloidin channel image (propagation method using minimum cross-entropy thresholding). Then, log10(cellular area/nucleus area) was calculated per object and the average per well was used for further quantification.

### Statistics.

Data analysis and figure preparations were done using GraphPad Prism 9.5.1 (for bar graphs and stack plots) and Excel. Statistical analysis was performed in GraphPad Prism 9.5.1. The individual statistical methods used are indicated in the figures. A *P* value < 0.05 was considered significant.

### Data availability.

Data are available in the Supporting Data Values file and from the corresponding author on request.

## Author contributions

AM performed the main experiments and analysis, interpreted the data, and wrote the manuscript. RK and JDS performed mouse experiments, analyzed samples, and reviewed the manuscript. KB identified the patient, collected patient specimens, and identified the mutation. FB analyzed the impact of SPLIS mutations on SPL structure. TH supervised the study and edited and critically revised the manuscript.

## Conflict of interest

JDS is a cofounder of Sphinxion Therapeutics, Inc., which is focused on developing therapeutics for SPLIS. She is an inventor on 3 patents related to development of gene therapy and blood biomarkers for SPLIS.

## Funding support

This work is the result of NIH funding, in whole or in part, and is subject to the NIH Public Access Policy. Through acceptance of this federal funding, the NIH has been given a right to make the work publicly available in PubMed Central.

- NIH U2C/TL1 LAUNCH program (National Institute of Diabetes and Digestive and Kidney Diseases grant 5TL1DK139565-03 to RK).
- NIH (grants R21OD037868-01 and R01HD113778 to JDS).
- California Institute of Regenerative Medicine (grant DISC2-13072 to JS).
- The RTW Charitable Foundation (to JDS).
- Harrington Discovery Institute (to JDS).
- The Swiss National Science Foundation (SNF) (grant SNF 310030_215134 to TH) and by the SNF under the frame of the European Joint Program on Rare Diseases (grant 32ER30_187505 to TH).

## Supplementary Material

Supplemental data

Supporting data values

## Figures and Tables

**Table 1:**
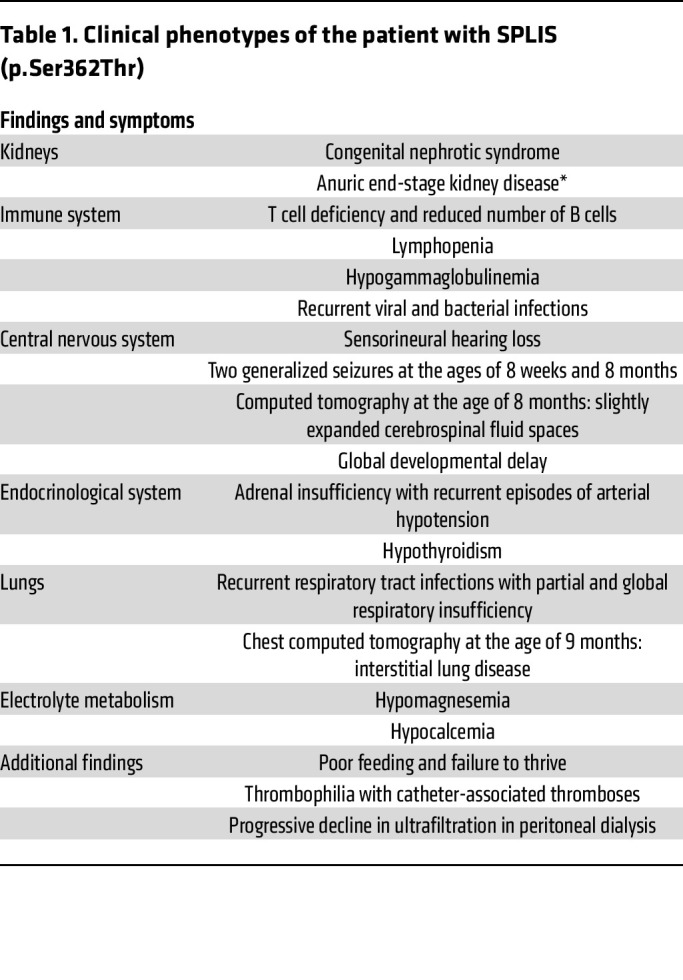
Clinical phenotypes of the patient with SPLIS (p.Ser362Thr)

**Table 2:**
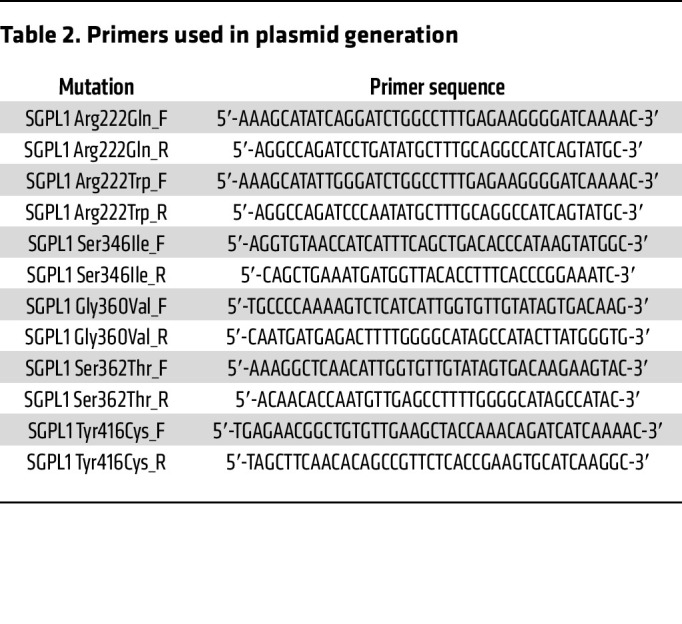
Primers used in plasmid generation

## Version 1 04/23/2026

In-Press Preview

## Version 2 06/15/2026

Electronic publication
