# Supplementary material for: Redirection of sphingolipid metabolism drives cytoskeletal defects in SPLIS and reveals ROCK inhibition as therapy
Source: J Clin Invest. 2026 Apr 23;136(12):e194427. doi: 10.1172/JCI194427 (PMC13262714; doi:10.1172/JCI194427)
Supplement: Supplemental data [file jci-136-194427-s067.pdf]

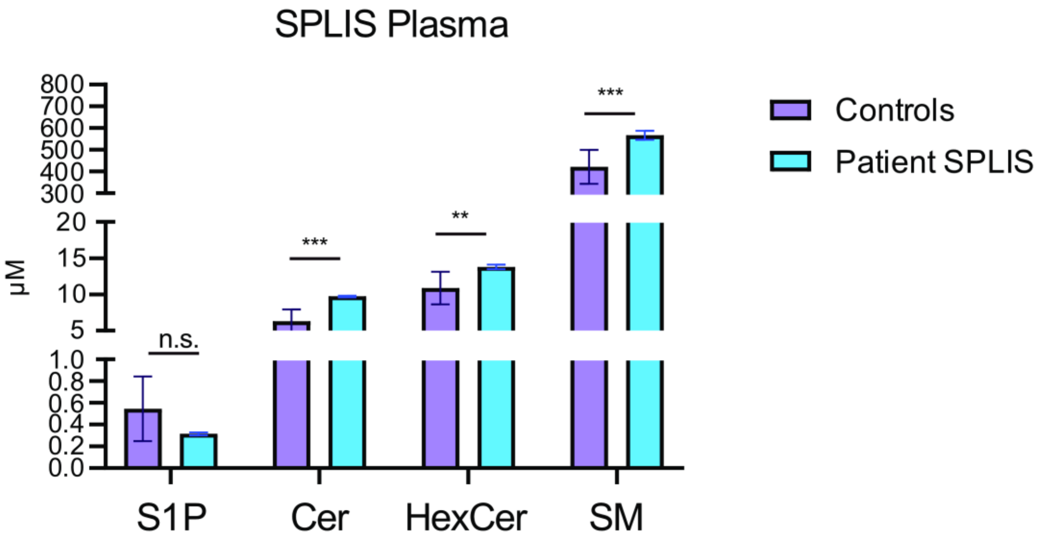

Supplementary Figure 1 Plasma sphingolipid (SL) profile of the SPLIS (p.Ser362Thr) patient (N=1 with three technical replicates) compared to healthy controls (N=7).

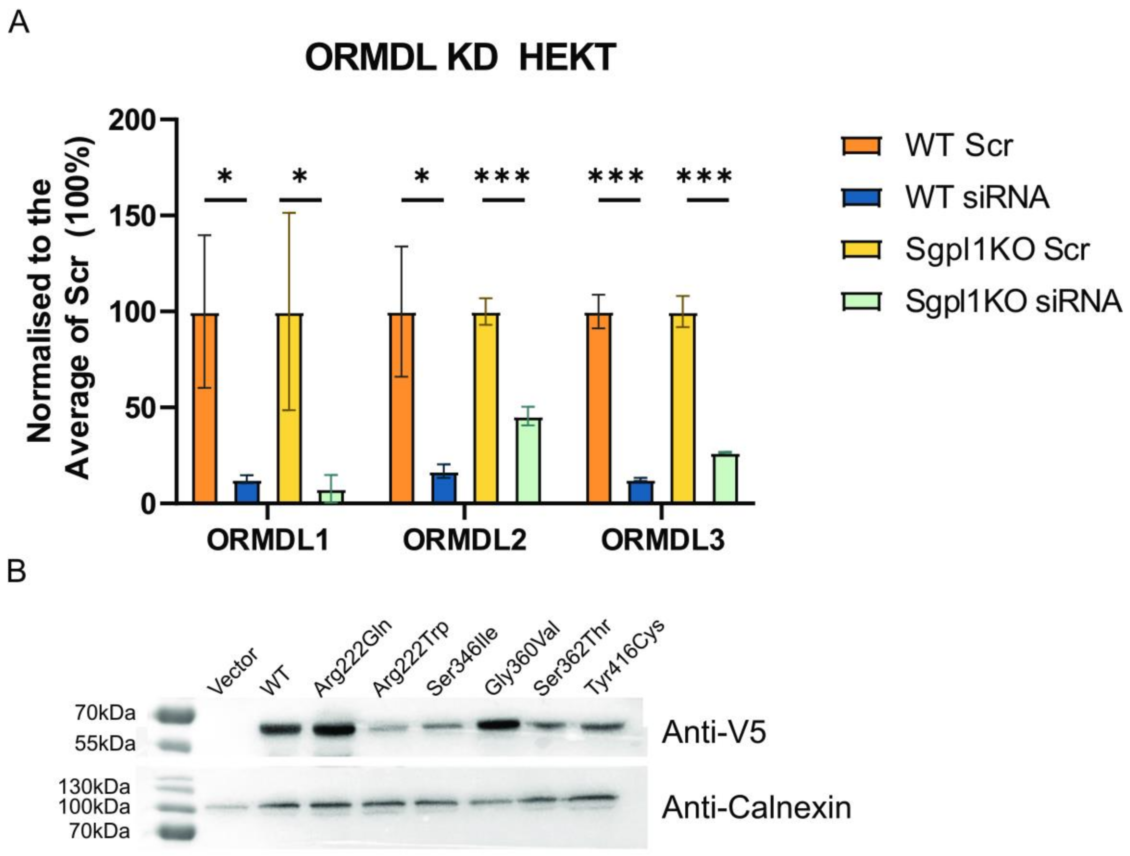

Supplementary Figure 2 (A) ORMDL expression after knockdown (KD) with ORMDL 1, 2, 3, siRNA or non-coding Scramble (Scr) as quantified by qRT-PCR. Total RNA levels were calculated from a dilution curve and normalized to GAPDH. Levels were normalized to Scr. In total three tests were performed. Multiple testing was corrected using two-stage step-up method (Benjamini, Krieger and Yekutieli). *=q<0.05, **=q<0.005, ***=q<0.0005.

(B) Expression of the different SPL mutants in an HEKT *SGPL1* KO background. pLenti-beta-Galactosidase-V5 expressed in HEKT *SGPL1* KO was used as Vector control. Anti-Calnexin was used as loading control

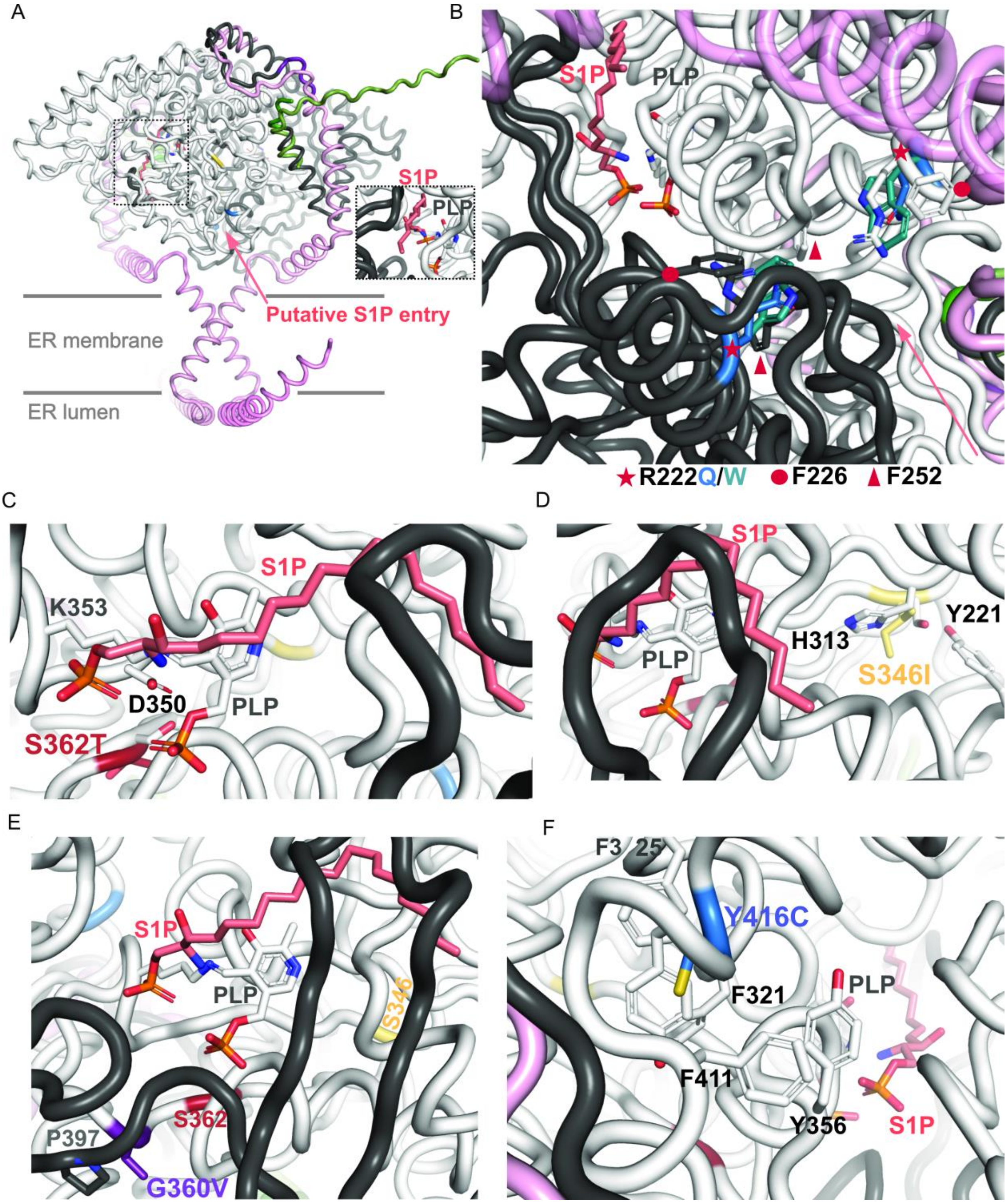

Supplementary Figure 3. Structural analysis of human SPL and effects of disease-associated mutations.

(A) SPL dimer structure. Subunits are white and dark grey; N- and C-termini are shown in violet and dark green. The arrow indicates the putative entry channel guiding S1P to the active site. Insert: active site containing the internal aldimine (PLP) and S1P.

(B) Close-up of the entry gate formed by residue R222. Positively charged R222 residues from both subunits repel each other, keeping the S1P entry channel open and guiding S1P to the active site. The R222N mutation may partially close this gate through dipole–dipole interactions, slightly reducing S1P entry. In contrast, the R222W mutation likely closes the gate via long-range hydrophobic interactions. Additionally, S1P may interact with hydrophobic clusters formed by W222, F226, and F252, slowing its accommodation into the active site.
(C) Catalysis is strongly impaired in mutant S362T, located 9 Å from PLP and 3.0 Å from D350. In silico modelling with WinCoot required swapping φ and ψ torsion angles to fit T362, indicating structural strains. In vivo, the S362T substitution may displace K353 of the internal aldimine and impair PLP binding, consistent with the patient phenotype (Table 2) and S1P accumulation (Fig. 4D). **(D)** Mutation S346I disrupts the dipole–dipole interaction with H313. H313 likely shifts towards PLP, reducing S1P binding and slowing catalysis. PLP binding may also be affected because residue 346 lies on the same loop as S362. Interaction between I346 and Y221 may further influence opening of the R222 entry gate.
(E) G360, located on the same loop as S362 and S346, is mutated in G360V. This likely causes a milder effect than S362T due to greater distance from PLP, although P397 from the second subunit rigidifies the loop and pushes V360 towards PLP.
(F) Y416 lies within a hydrophobic cluster (F411, F321, F325, Y356) ~15 Å from PLP that may facilitate product release. Mutation Y416C disrupts this cluster, slowing product exit and contributing to S1P accumulation (Fig. 4D).
Residues 1–128 and 544–568 were modelled with AlphaFold3 (pTM = 0.87). Figures were generated in PyMOL 2.5.0; mutants were introduced and energy-minimized using WinCoot 0.9.8.92. (SPL; PDB: 8ayf)
